# Comparison of Aerobic Scope for Metabolic Activity in Aquatic Ectotherms With Temperature Related Metabolic Stimulation: A Novel Approach for Aerobic Power Budget

**DOI:** 10.3389/fphys.2018.01438

**Published:** 2018-10-22

**Authors:** Kurt Paschke, José Agüero, Paulina Gebauer, Fernando Díaz, Maite Mascaró, Estefany López-Ripoll, Denisse Re, Claudia Caamal-Monsreal, Nelly Tremblay, Hans-Otto Pörtner, Carlos Rosas

**Affiliations:** ^1^Instituto de Acuicultura, Universidad Austral de Chile, Puerto Montt, Chile; ^2^Centro FONDAP de Investigación en Dinámica de Ecosistemas Marinos de Altas Latitudes (IDEAL), Punta Arenas, Chile; ^3^Posgrado en Ciencias del Mar y Limnología, Universidad Nacional Autónoma de México, Ciudad de Mexico, Mexico; ^4^Centro i~mar, Universidad de Los Lagos, Puerto Montt, Chile; ^5^Laboratorio de Ecofisiología de Organismos Acuáticos, Departamento de Biotecnología Marina, Centro de Investigación Científica y de Educación Superior de Ensenada, Ensenada, Mexico; ^6^Unidad Multidisciplinaria de Docencia e Investigación, Facultad de Ciencias, Universidad Nacional Autónoma de Mexico, Sisal, Mexico; ^7^Laboratorio de Resiliencia Costera (LANRESC, CONACYT), Sisal, Mexico; ^8^Alfred Wegener Institute Helmholtz Centre for Polar and Marine Research, Biologische Anstalt Helgoland, Shelf Seas Systems Ecology, Helgoland, Germany; ^9^Alfred Wegener Institute, Helmholtz Centre for Polar and Marine Research, Integrative Ecophysiology, Bremerhaven, Germany

**Keywords:** metabolic scope method, metabolism, lactate, thermal tolerance, metabolic rate, sluggish fish

## Abstract

Considering that swim-flume or chasing methods fail in the estimation of maximum metabolic rate and in the estimation of Aerobic Scope (AS) of sedentary or sluggish aquatic ectotherms, we propose a novel conceptual approach in which high metabolic rates can be obtained through stimulation of organism metabolic activity using high and low non-lethal temperatures that induce high (HMR) and low metabolic rates (LMR), This method was defined as TIMR: Temperature Induced Metabolic Rate, designed to obtain an aerobic power budget based on temperature-induced metabolic scope which may mirror thermal metabolic scope (TMS = HMR—LMR). Prior to use, the researcher should know the critical thermal maximum (CT max) and minimum (CT min) of animals, and calculate temperature TIMR max (at temperatures −5–10% below CT max) and TIMR min (at temperatures +5–10% above CT min), or choose a high and low non-lethal temperature that provoke a higher and lower metabolic rate than observed in routine conditions. Two sets of experiments were carried out. The first compared swim-flume open respirometry and the TIMR protocol using *Centropomus undecimalis* (snook), an endurance swimmer, acclimated at different temperatures. Results showed that independent of the method used and of the magnitude of the metabolic response, a similar relationship between maximum metabolic budget and acclimation temperature was observed, demonstrating that the TIMR method allows the identification of TMS. The second evaluated the effect of acclimation temperature in snook, semi-sedentary yellow tail (*Ocyurus chrysurus*), and sedentary clownfish (*Amphiprion ocellaris*), using TIMR and the chasing method. Both methods produced similar maximum metabolic rates in snook and yellowtail fish, but strong differences became visible in clownfish. In clownfish, the TIMR method led to a significantly higher TMS than the chasing method indicating that chasing may not fully exploit the aerobic power budget in sedentary species. Thus, the TIMR method provides an alternative way to estimate the difference between high and low metabolic activity under different acclimation conditions that, although not equivalent to AS may allow the standardized estimation of TMS that is relevant for sedentary species where measurement of AS via maximal swimming is inappropriate.

## Introduction

The aerobic power budget, often measured as aerobic scope (AS), is the surplus proportion of the energy flux (and the corresponding amount of metabolic power used to support this flux) that is left after the basal maintenance costs of an organism are met. It reflects the energy that an individual can invest into somatic and gamete production, and other fitness-related functions (Guderley and Pörtner, 2010; Sokolova et al., 2012). In practice, AS is calculated as the difference between the maximum metabolic rate (MMR) and the standard metabolic rate (SMR), the latter being a widely used indicator of the minimal rate of energy required to maintain life. According to the concept underlying the “oxygen and capacity limitation of thermal tolerance” OCLTT; (Pörtner, 2002, 2010) the ability (or lack thereof) to sustain aerobic scope emerged as a major criterion to distinguish between moderate and extreme thermal stress (Sokolova et al., 2012).

In a recent special issue of the Journal of Fish Biology, the multi-national group of experts that forms the Cooperation in Science and Technology-Action Conservation Physiology of Marine Fishes (COST FA1004) recognized that the methods most used for the evaluation of the maximum metabolic rate (MMR), being chase or swimming funnel, do not work well in all species, indicating that the applicability of various methods also depends on the athletic characteristics of each fish species (Chabot et al., 2016a; Norin and Clark, 2016). These authors emphasized that MMR measurements depend on intrinsic factors related to the way in which each species reacts to maximum activity, as well as on environmental factors affecting the animal's metabolism. While the ecological relevance of MMR is controversial (Clark et al., 2013; Deutsch et al., 2015; Farrell, 2016; Norin and Clark, 2016), results obtained for different aquatic ectotherms suggest that steady state MMR measured in continually active species, such as salmon migrating upstream, is a good indicator of physiological plasticity and could be used to assess exposure to environmental stressors (Eliason et al., 2011; Sokolova et al., 2012; Rummer et al., 2014; Farrell, 2016).

The concept of AS is very much tied to the measurement of MMR during exercise protocols. Two methods are widely accepted and commonly used to determine the MMR in fish: (i) the swim-flume respirometry protocol, based on the critical swimming speed (Brett, 1964; Eliason and Farrell, 2016) and (ii) manual chasing for 5 min (Roche et al., 2013; Norin et al., 2014; Norin and Clark, 2016). Both swim-flume and chasing are designed to measure the MMR of fish or invertebrates during or immediately after intense and exhaustive exercise (Brett, 1964; Roche et al., 2013; Norin et al., 2014; Ern et al., 2015). The basic concept behind those measurements is that when the difference between MMR and SMR is calculated, the resulting curve (AS) gives a quantitative description of the aerobic capacity of the animal, allowing the identification of optimal (resulting in maximum AS) and stressful (resulting in reduced AS) environmental conditions (Fry, 1947, 1971; Brett, 1964). However, results obtained using these two methods do not always coincide. In the coral reef fish *Scolopsis bilineata*, MMR values were lower using the chasing method than with swim-flume respirometry, and it was suggested that such differences could be explained by the variability intrinsic to the chase protocol (Roche et al., 2013). Generally, these two protocols successfully evaluate the metabolism of active fish, but other vertebrates and many invertebrates do not respond well to both MMR protocols because of their naturally low activity levels (Norin and Clark, 2016; Gvoždík and Kristín, 2017).

In sedentary animals or in those that do not display athletic swimming behavior, the most frequently used method is manual chasing, which consists of touching, pinching or using air exposure in an attempt to provoke maximum activity in the specimen (Ern et al., 2014, 2015; Norin et al., 2014). This method induces a high metabolic rate, but it is difficult to ensure that all experimental animals are receiving a chase stimulus of similar magnitude. The main problem arising from MMR measurements using chasing protocols is the low probability of obtaining comparable results because of the variability associated with the application of the protocol (Ern et al., 2014, 2015). In addition, when manual chasing is applied, there is a risk of injury (Ern et al., 2014) that modifies the physiological condition of the animal and, in consequence, affects MMR values. Standardized protocols to measure MMR are thus needed for sedentary ectotherms. Furthermore, MMR is usually measured post-exercise in sedentary ectotherms, during initial recovery from the chasing protocol. Other less standardized methods were used in sedentary organisms to measure MMR and calculate AS: manual chasing for 15 min (Roche et al., 2013), placing fish in a cylinder exposed to water current using magnetic stirring (Rummer et al., 2014), feeding New Zealand Geoduck clam with microalgae (Le et al., 2016, 2017) or feeding newts until they reach maximum metabolic rate (Gvoždík and Kristín, 2017).

To obtain AS, both MMR and SMR are necessary (Fry, 1947; Norin et al., 2014; Chabot et al., 2016b; Claireaux and Chabot, 2016); however, the evaluation of SMR represents a challenge due to ectotherm physiology. The experimenter should guarantee that the animals are at a strictly basal stage, *i.e.*, without any internal (digestion, growth, immune response, etc.) or external factors (noise, water flow, environmental changes, etc.) affecting metabolic rate (Chabot et al., 2016a). Because keeping animals at a strictly basal stage is unfeasible, many researchers have been using 24 h fasted animals in a respirometry chamber to obtain the minimum metabolic rate, which has been considered close to SMR (Norin et al., 2014; Rummer et al., 2014; Ern et al., 2015; Norin and Clark, 2016). Although there is a list of several recommendations to recognize when SMR has been reached, most studies report values that are somewhere between SMR and routine metabolic rate (RMR) (Chabot et al., 2016a).

Further proxies may be useful to estimate aerobic power budget. Fry (1947) defined temperature as a controlling factor because it governs maximum and minimum metabolic rates. Later on, it was shown in ectotherms that a strong and predictable relationship between activity and temperature results from molecular activation of the components of the metabolic chains (For review see Fry, 1971; Farrell, 2016). In fact, it is widely acknowledged that “activity is fundamentally the result of transformation of energy from one form to another and the application of that energy to a given performance” (Fry, 1971).

As a new conceptual approach, we propose measuring weight specific oxygen consumption when it is stimulated to provoke high metabolic rates (HMR), *i.e.*, when ectotherms are exposed to a temperature high enough to stimulate maximum metabolic rate for 5 min (Temperature that induced maximum metabolic rate: TIMR max). According to our trials with invertebrate species (Rodríguez-Fuentes et al., 2017), TIMR max in most of the organisms was seen at temperatures between 5 and 10% below CT max. Similarly, a minimum metabolic rate can be obtained when the activity is depressed for 5 min by exposure to a temperature low enough to provoke a forced low metabolic rate (LMR) (Temperature that induced resting metabolic rate: TIMR min; TIMR min is set to temperatures 5–10% above CT min). The 5 min duration is long enough to provoke the activation of respiratory metabolism, but short enough to avoid animals to experience harmful effects from heat exposure. Laboratory experiments have demonstrated that specimens regain routine metabolic rate 20–30 min after the TIMR max evaluation (Rodríguez-Fuentes et al., 2017).

**Figure 1:**
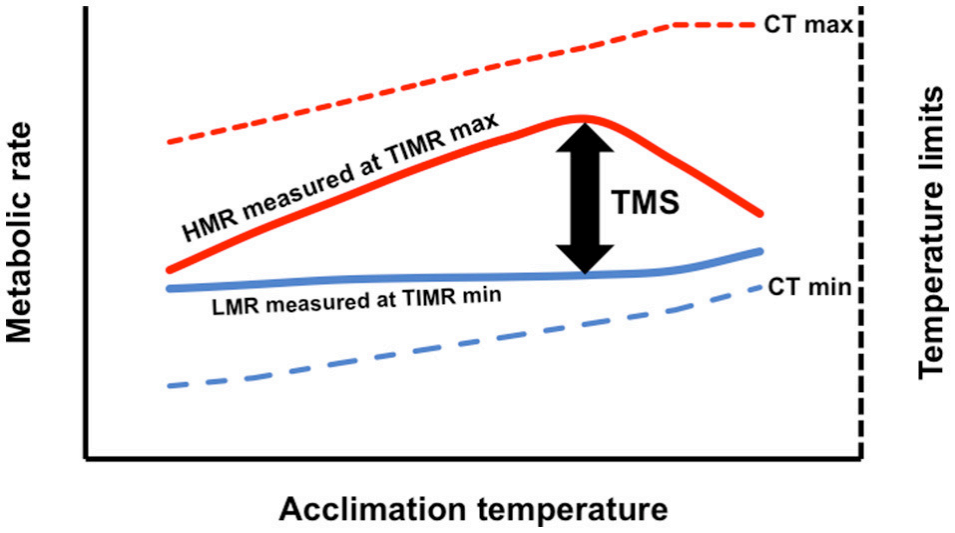
Conceptual model of temperature-induced metabolic rate method (TIMR) when aquatic ectotherms are acclimated at different temperatures. Thermal metabolic scope (TMS) is calculated as the difference between the high metabolic rate (HMR) and low metabolic rate (LMR) (Left axis). The temperatures used to induce metabolic rates min (TIMR min) and max (TIMR max) are 5–10% below or above the critical thermal minimum (CT min) or maximum (CT max) (Right axis).

Although LMR is not conceptually equivalent to SMR, by estimating a standardized low metabolic rate, we think that this oxygen consumption value can be used to calculate the thermal metabolic scope of non-athletic organisms in a similar way to when the minimum metabolic rate is obtained in an animal maintained 24 h in a respirometric chamber without disturbance (Chabot et al., 2016b). We hypothesize that a thermal metabolic scope (TMS), obtained by temperature-induced metabolic stimulation as the difference between HMR and LMR, could be used as a new indicator of the aerobic power budget for sedentary or sluggish organisms (Figure 1).

As it can be expected, higher temperatures increase energy demand due to rising maintenance costs (e.g., transmembrane ion exchange), and enhance the capacity of biochemical reactions involved in mitochondrial energy production (including oxygen consumption), allowing more energy to be produced and invested in performances. Consequently, in this study, temperature was used with a two-fold aim: as an experimental factor (acclimation period), and as a tool to induce analogs of low (LMR) and high metabolic rates (HMR) that can be used as indicators of ectotherm performance. With the TIMR approach, oxygen consumption measurements can be obtained fast enough to avoid the activation of compensatory mechanisms, but slow enough for animals to respond only to temperature changes used during the evaluations (*i.e.*, 5 min short-term exposure). This approach was developed to consider organisms that cannot be studied using the conventional swim-flume or chase methods, using temperature increments to maximize stimulation of aerobic metabolism, without potential mechanical damage.

To compare the TIMR method with those more frequently used (swim-flume and chasing), we used fish as models in two sets of experiments, taking into consideration that fish are the most studied group of ectothermic aquatic organisms. The first was designed to compare the TIMR method and swim-flume respirometry using *Centropomus undecimalis* (snook), an endurance swimmer, acclimated to 28 (optimal temperature), and 32 and 35°C (sub-optimal temperatures) (Noyola Regil et al., 2015). Snook distribution and movements can be driven by spawning (they are obligate marine spawners) or prey availability, reaching large rivers to capitalize on prey (Trotter et al., 2012). Considering the manner in which each method stimulates fish metabolic activity (Fry, 1971; Lurman et al., 2007), swim-flume and TIMR methods were compared to establish whether the form in which acclimation temperatures modulate metabolic performance is similar, regardless of the different magnitude of the responses observed in this athletic fish species.

The second set of experiments evaluated the effect of long-term (21 days) acclimation temperature on metabolic scope in snook, yellow tail (*Ocyurus chrysurus*), and clownfish (*Amphiprion ocellaris*), three fish with different swimming behaviors. Yellow tail has been classified as common transient predator that frequently moves from refuges (in close mangroves or under the reef) to reef patches where prey are abundant, while the clownfish lives linked to an anemone where they obtain food and protection (Madeira et al., 2016b; Harborne et al., 2017). TIMR and chase methods were used to test the hypothesis that with both methods, it is possible to identify the thermal window of fish and the dependence of metabolic scope on acclimation temperature, independent of differences in their swimming behavior.

Blood lactate levels were included as an indicator of the onset of anaerobic metabolism, to assess if swim-flume, chase, and TIMR methods provoke an anaerobic metabolic response in experimental animals. This would also indicate whether exhaustion or temperature induced constraints reach the limits of aerobic metabolic activity. Finally, the advantages and disadvantages of the TIMR method were analyzed, in an attempt to encourage a wider use of this novel approach to evaluate the aerobic power budget of aquatic species that are immobile or with very little movement, among sedentary invertebrates and non-athletic fish.

## Materials and methods

### Origin of experimental animals

Juvenile common snook (*C. undecimalis*) (*n* = 168; 39 ± 11 g wet weight WW; mean ± SD) and yellow tail *(O, chrysurus*) (*n* = 118; 9.15 ± 3.17 g WW) were obtained from aquaculture research facilities located at UNAM in Sisal (Yucatán, Mexico), where housing and feeding conditions were previously established (Ibarra-Castro et al., 2011). Once transferred to the laboratory, fish were placed in 60 L plastic tanks (12 individuals per tank) in a temperature controlled (26 ± 1°C), filtered and fully aerated seawater recirculation system. Approximately 5% of seawater in the system was exchanged every day. The snook were kept for 21 days under the following constant acclimation temperatures: 18, 22, 26, 28, 30, 32, 35, and 38°C. In the same manner, yellow tail fish were acclimated at 20, 22, 26, 30 and 32°C. During this period, all fish were manually fed *ad libitum* twice a day (09:00 and 17:00 h) with a commercial diet (Marubeni Nisshin Feed Co., Ltd., Japan).

Clownfish were obtained from laboratory production of ornamental fish of the Center for Food Research and Development, AC (CIAD) located in Mazatlan (Sinaloa, Mexico). The organisms were transported by air, to the laboratory of Marine Biotechnology CICESE, where 540 juveniles (*n* = 540; range of 0.7–2.5 g WW) were placed in 6 tanks of 200 L at 26 ± 1°C for 9 days. Afterwards, the fish were conditioned to experimental temperatures of 20, 23, 26, 29, 32 and 35°C. To reach those temperatures, a rate of 2°C per day was used. The fish remained at these experimental temperatures for 21 days. The clownfish were fed a commercial diet (Skretting 0.8 mm) at 5% of their wet weight twice a day.

In all cases, acclimation temperature was controlled (± 0.3°C) using titanium chillers and submersible titanium heaters connected to digital controls with a thermocouple. Animals were maintained under a 12 h light: dark photoperiod and were fasted for 24 h prior to any experimental trials to ensure they were in a post-absorptive state (Beamish, 1978). In order to avoid violations of the statistical principle of independence, different and randomly selected individuals were used in every treatment and level (Horodysky et al., 2011).

### Experiment 1: swim-flume respirometry (only snook)

Both routine metabolic rate (RMR) and maximum metabolic rate (MMR) were determined by assessing the swimming performance of individual fish in a custom built 1.65 L clear acrylic, open (flow-through) swim-flume respirometer, measuring 7.0 cm in width and 41.0 cm in length (Supplemental Video 1). A honeycomb pattern was used to create laminar flow within the working section and uniform water flow was confirmed using the dye technique. The respirometer was coupled to a temperature-controlled, filtered and well-aerated seawater recirculation system (Supplemental Video 1).

The rate of seawater flow to be used for *C. undecimalis* swimming experiments was determined before the first experiment took place. Fish acclimated to 30°C were individually placed in the swim-flume at a velocity of 2 cm s^−1^ (*N* = 15) during a conditioning period of 30 min in a semi-dark and quiet environment. This allowed the fish to rest (Tolley and Torres, 2002; Tierney, 2011) and stable oxygen consumption measurements were obtained. After the conditioning period, the flow was quickly increased to 4 (*N* = 5), 9 (*N* = 5), and 13 (*N* = 5) cm s^−1^ for 5 min, as recommended by Norin and Clark (2016). Different fish were tested at each velocity to obtain independent oxygen consumption data (Horodysky et al., 2011). Valves regulated water flow rates in the flume, and water velocity was calculated by applying the equation (1) for a known volume of water (*i.e.*, 1,650 cm^3^):

(1) $$\begin{array}{r} v=\frac{V}{\pi r^{2}\cdot t} \end{array}$$

where *v* is water velocity (cm s^−1^), *V* is a known volume of water (cm^3^), *r* is the radius of the flume, and *t* is time (s).

Oxygen concentration was measured every 15 s at the inflow and outflow with a Flow-Through oxygen sensor (Loligo Systems, Copenhagen, Denmark) connected to a PC-controlled fiber optic trace oxygen transmitter with temperature correction (OXY-10 trace Transmitter, PreSens Precision Sensing GmbH, Regensburg, Germany). Prior to each trial, the flow-through cell O_2_ sensor was calibrated at each acclimation temperature using air-saturated seawater (100%) and with 1–1.5% anhydrous sodium sulfite (at 0%). According to the manufacturer, sensor drift is < 0.15% O_2_ at 0 oxygen and < 0.475% O_2_ at air saturation (Loligo systems, Copenhagen, Denmark). Oxygen consumption rates (mg O_2_ h^−1^ g WW^−1^) were calculated as shown in equation (2):

(2) $$O_{2}\text{ consumption}=V_{W}\cdot \frac{\left(C_{W}O_{2\;in}-C_{W}O_{2\;out}\right)}{M}$$

where *V*_*W*_ is water flow through the respirometer (L h^−1^), *C*_*W*_*O*_2 *in*_ is oxygen concentration of inflowing water (mg O_2_ L^−1^), *C*_*W*_*O*_2 *out*_ is oxygen concentration of outflowing water (mg O_2_ L^−1^), and *M* is body mass of experimental animals (g WW). Data were recorded once the oxygen concentration in both inflowing and outflowing waters was stable.

**Figure 2:**
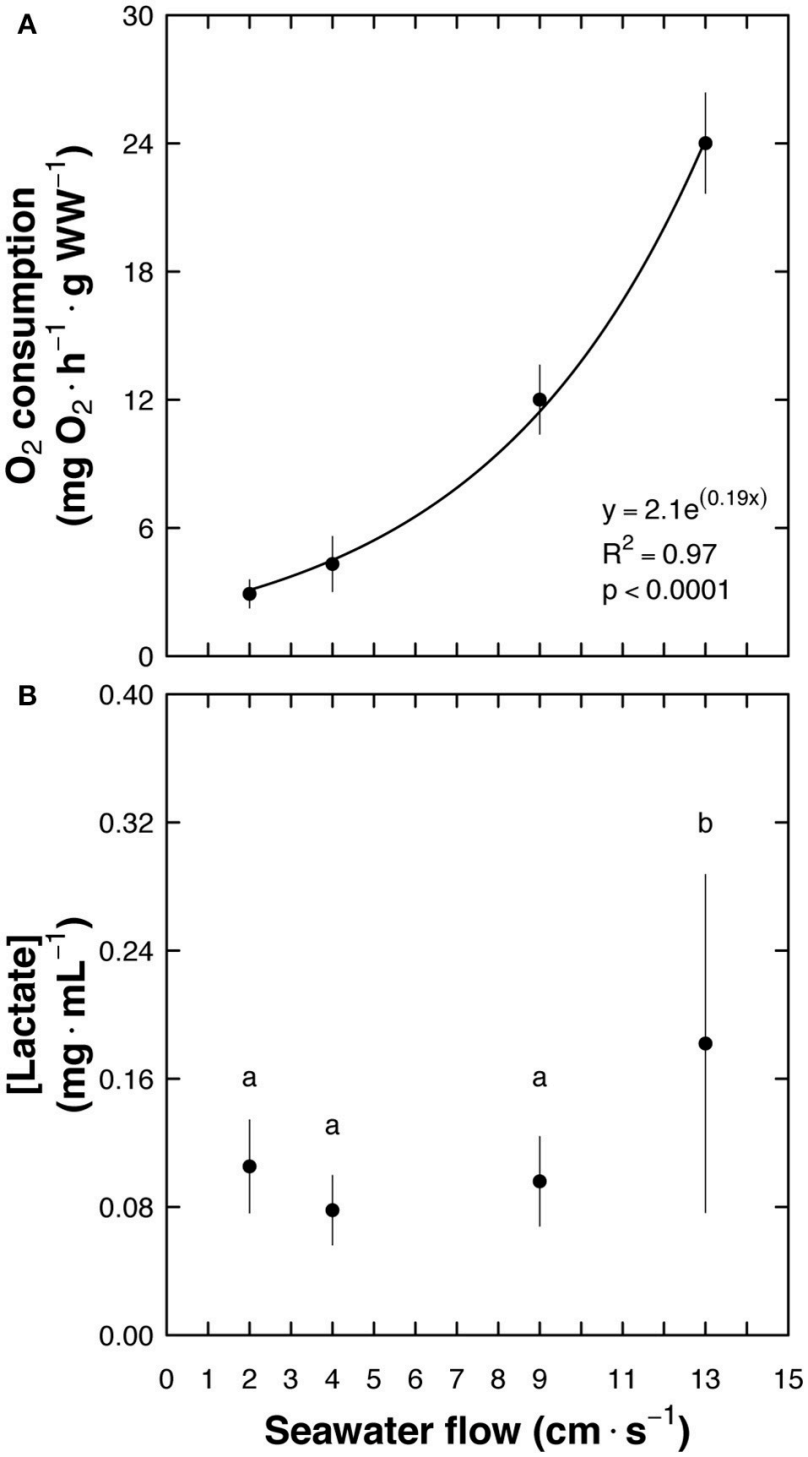
*Centropomus undecimalis* (snook) acclimated at 30°C and exposed to different seawater flows in a swim-flume respirometer. **(A)** Oxygen consumption (mg O_2_ h^−1^ g WW^−1^) at different flows (cm s^−1^; *n* = 5 at each flow) and **(B)** blood lactate levels (mean ± SD; mg mL^−1^) after the trial. Note that when animals were forced to swim in a seawater flow of 13 cm s^−1^ lactate values increased significantly in comparison with those obtained in animals swimming between 2 and 9 cm s^−1^ seawater flow.

The maximum water flow that *C. undecimalis* could endure for 5 min was 13 cm s^−1^. When the water flow was increased to 17 cm s^−1^, fish were unable to maintain their position in the swim chamber and were forced to rest against the back grid of the respirometer. A relationship between oxygen consumption and water flow was obtained, where O_2_ consumption = 2.1e^0.19×^ (*p* < 0.0001; Figure 2A). The penultimate water flow (9 cm s^−1^) before the fish fatigued and stopped swimming was used as the criterion to determine MMR, taking into consideration that exhaustion is the point when aerobic and anaerobic resources may have been expended as seen with lactate levels at 13 cm s^−1^ (*F* = 14.07; *p* < 0.0001) (Brett, 1964; Lurman et al., 2007) (Figure 2B). Routine metabolic rate (RMR) was defined as the oxygen consumption of fish in the swim-flume respirometer, measured in water flowing at a rate of 2 cm s^−1^, during 24 h (Tolley and Torres, 2002; Norin and Clark, 2016) (Supplemental Video 1). This water flow speed was used considering that snook as an athletic fish swims continuously, even at low temperatures.

Once the seawater flow to be used in the swim-flume was determined, the effects of temperature acclimation were tested using snook acclimated to temperatures of 28, 32, and 35°C, testing them at their respective acclimation temperatures. Individual fish (*n* = 6 per temperature) were placed in the swim-flume respirometer and kept in water flowing at 2 cm s^−1^ during a 24 h conditioning period. The water flow was then quickly increased to 9 cm s^−1^ and was maintained at this velocity for 5 min. Oxygen consumption recorded after 5 min of maximum activity was considered MMR (Norin and Clark, 2016). Following convention, aerobic scope (AS) was defined and calculated as the difference between MMR and SMR which was considered to be reflected by the minimal RMR obtained at 2 cm s^−1^ during 24h in the swim-flume respirometer (Chabot et al., 2016a,b; Norin and Clark, 2016).

### Experiment 2: chase protocol and temperature-induced metabolic rate method

#### Chase protocol

Six individual fish from each acclimation tank were rapidly and gently netted and placed in a circular exercise tank (47 cm diameter, 15 cm water depth) containing temperature-controlled and well-aerated seawater. Fish were given 15 min to recover from handling before they were continuously chased with a net for 5 min. The fish were lightly touched on the tail fin only if they slowed down or stopped swimming (Roche et al., 2013; Norin et al., 2014) (Supplemental Video 2). The same person chased all fish in a similar manner to prevent observer variations. After exercise, fish were immediately placed in a closed respirometry chamber submerged in a temperature controlled seawater bath, where post-exercise oxygen consumption was measured for 5 min following the standard procedures used in several fish species (Norin et al., 2014; Norin and Clark, 2016) (Supplemental Video 3). Initial and final oxygen concentrations were measured with a polarographic dissolved oxygen meter (YSI Pro20 Instrument, YSI Incorporated, Yellow Springs, OH, USA) previously calibrated at each experimental temperature. Taking into consideration that this oxygen sensor takes 30–45 min to stabilize after temperature changes, we calibrated the sensor and kept it in the same experimental temperature for 1 h before the oxygen consumption measurements (Ern et al., 2014, 2015). Three chambers filled with water without fish were used as controls to measure the oxygen consumed by microorganisms. Oxygen consumption rate (mg O_2_ h^−1^ g WW^−1^) was calculated as shown in equation (3):

(3) $$\begin{array}{r} O_{2}\text{ consumption}=\left(O_{2\left(A\right)}-O_{2\left(B\right)}\right)\cdot \frac{\left(V/t\right)}{M} \end{array}$$

where *O*_2(*A*)_ is the initial oxygen concentration in the chamber (mg O_2_ L^−1^), *O*_2(*B*)_ is the final oxygen concentration in the chamber (mg O_2_ L^−1^), *V* is the water volume in the chamber minus the volume of water displaced by the animal, *t* is the time elapsed during measurement (h), and *M* is body mass of the experimental animal (g WW). Oxygen consumption values obtained after the chase protocol were considered the MMR of fish.

Taking into consideration that the evaluation of SMR requires minimal activity of the fish (Chabot et al., 2016b), we were more conservative using the term RMR to assess the calculation of AS. The RMR was identified as the lower 20th percentile of the recorded oxygen consumption values obtained in fish maintained for 24 h in a flow-through respirometer at each acclimation temperature (Chabot et al., 2016b; Rodríguez-Fuentes et al., 2017). RMR was measured using a continuous flow respirometer, with the respirometric chambers connected to a well-aerated re-circulating seawater system at the acclimation temperature. Measurements of dissolved oxygen (mg L^−1^) were recorded for each chamber (entrance and exit) every second using oxygen sensors attached to flow-cells that were connected by optical fiber to a witrox amplifier (Loligo Systems, Denmark). A chamber without fish was used as a control to account for microbial oxygen consumption in the filtered seawater. The sensors were previously calibrated at each temperature using saturated seawater (100% air saturation) and a 1% sodium sulfite solution (0% air saturation). RMR was calculated using equation 2 over 24 h. The respiration values used for comparison were selected using the R package “fishMO2” created by Chabot (2016) according to several methods exposed in Chabot et al. (2016b).

#### Temperature-induced metabolic rate (TIMR) method

Each trial started by moving a fish (snook, yellow tail or clownfish) rapidly and gently from its acclimation tank into a closed respirometry chamber submerged in a temperature-controlled seawater bath at TIMR min or TIMR max temperature for 5 min. For defining the respective temperatures CT values were previously analyzed for each species and acclimation temperature (Table 1). Temperatures to induce LMR (Supplemental Video 4) and HMR (Supplemental Video 5) were calculated as 5–10% lower or higher than CT max and CT min, respectively (Table 1). HMR and LMR were calculated using equation 3. In all measurements, oxygen concentration in the chamber remained always above 80% air saturation. Initial and final oxygen concentrations were measured for snook and yellow tail (*N* = 6 individuals) at each acclimation temperature with a polarographic oxygen meter (YSI Pro20 Instrument, YSI Incorporated, Yellow Springs, OH, USA) previously calibrated and stabilized for 1 h before measurement at each experimental temperature (Ern et al., 2014, 2015). Taking into consideration that TIMR max and min depend on the species and on the acclimation temperature, species specific CT max and min data have been compiled in (Table S1) to calculate TIMR max and min of molluscs (15 species), crustaceans (36 species) and fish (87 species). Recently, a list with the CT values of 2,133 species of multicellular algae, plants, fungi, and animals in 43 classes, 203 orders and 525 families from marine, intertidal, freshwater, and terrestrial realms, extracted from published studies was published (Bennett et al., 2018).

**Table 1:** Acclimation temperatures used to determine critical thermal maximum and minimum (CT max, CT min) at a rate of 1°C min^−1^ in three fish species.

	**Acclimation**	**Water-bath temperatures for TIMR determinations, °C**			
**Species**	**T°C**	**CT min**	**CT max**	**TIMR min**	**TIMR max**
*Centropomus undecimalis,*	18	11	37.0	12.1	33.3
(Noyola Regil et al., 2015)	22	11	37.9	12.1	34.1
	26	11.8	38.3	13.0	34.5
	28	13	40.3	14.3	36.2
	30	13.1	41.2	14.4	37.0
	32	15	41.0	16.5	36.9
	35	17	43.0	18.7	38.7
	38	NA	NA	NA	38.7^a^
*Ocyurus chrysurus*	20	11.5	36.7	12.6	33
(Noyola Regil et al., 2015)	22	11.8	37.7	13	33.9
	26	14.8	37.8	16.3	34
	30	15.5	38.9	17	35
	32	16.4	40	18	36
*Amphiprion ocellaris^b^*	20	14.8	31.4	15.5	29.8
Unpublished data	22	16.5	32.5	17.3	30.9
	26	18.0	33.6	18.9	31.9
	30	20.2	35.7	21.2	33.9
	32	22.0	35.8	23.1	34.0
	35	23.7	36.0	24.9	34.2

Mean values of reported CT max and CT min were used to calculate the water-bath temperatures to which individuals should be exposed in order to induce high (TIMR max) and low (TIMR min) metabolic rates. TIMR max and TIMR min were calculated for 90% of CT max and 110% of CT min, respectively.

*NA, Not available*.

a *This temperature was used because snook exposed to higher temperatures died in the chamber*.

b *Clownfish TIMR tests were carried out at 95 and 105%, of CT max and min respectively, because at those temperatures animals showed a sustained higher activity and a lower activity during 5 min*.

**Figure 3:**
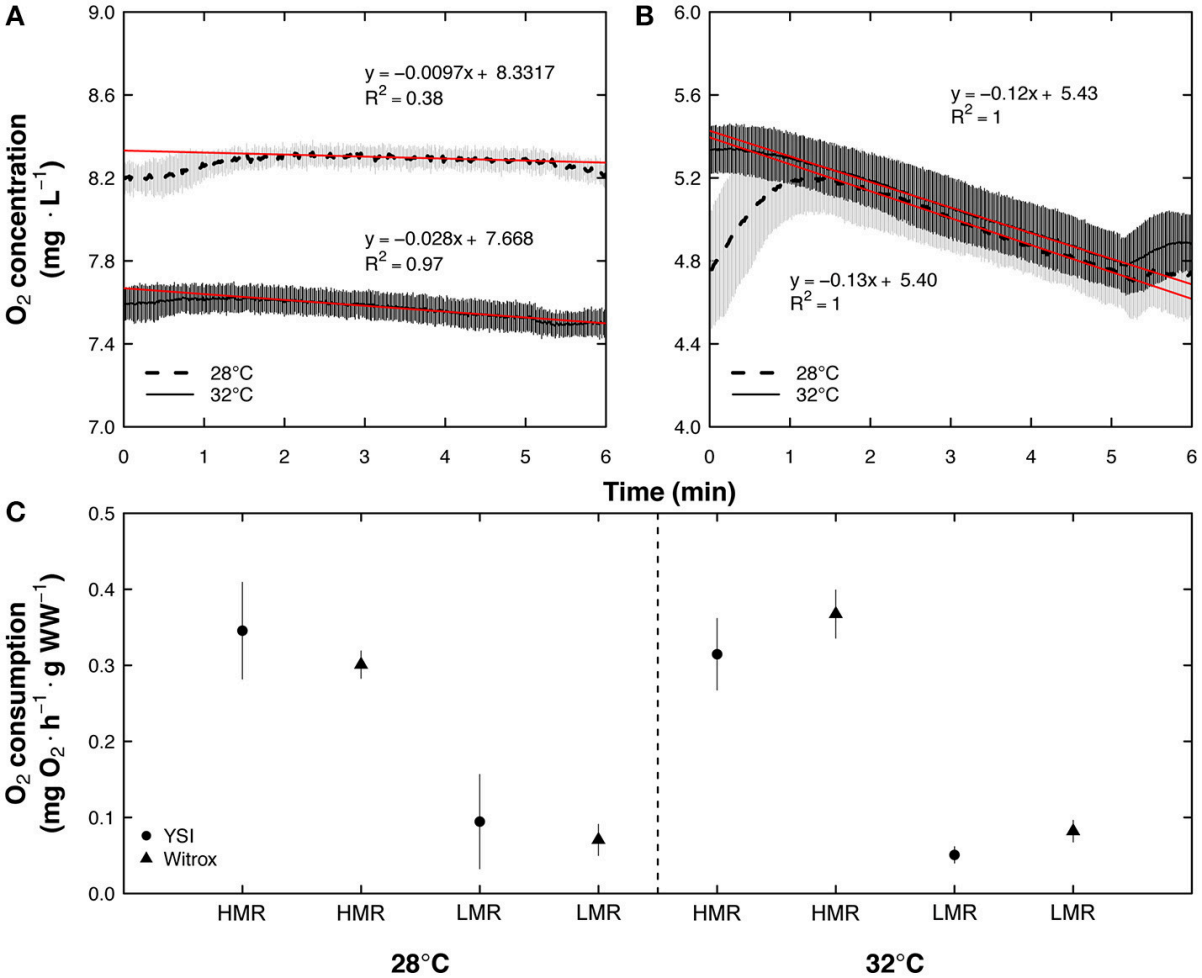
Temperature-induced metabolic rate (TIMR) measurements with PC-controlled fiber optic trace oxygen transmitter (Witrox) and a handheld dissolved oxygen meter (YSI) for *Centropomus undecimalis* acclimated at 28 and 32°C in a closed-system. **(A)** Measurements were made at low (110% CT min: TIMR min) and **(B)** high (90% CT max: TIMR max) temperatures for 23 C. *undecimalis* juveniles acclimated for 21 days at 28 (dashed line) and 32°C (full line). To calculate low metabolic rate (LMR at TIMR min) and high metabolic rate (HMR at TIMR max), the first and last minute of each slope was discarded due to the oxygen adjustments in the chamber when each fish was introduced and taken from the respirometric chamber, respectively. The background oxygen consumption obtained from control chambers without fish (*n* = 3 per TIMR and experimental temperature) was considered. HMR and LMR (mg O_2_ h^−1^ g^−1^ WW) measured with the optical sensor were calculated from the slopes of O_2_ consumed (red line). **(C)** These HMR and LMR (triangles; mean ± SD) were compared with those obtained from YSI instrument (circles; mean ± SD; mg O_2_ h^−1^ g^−1^ WW).

Results obtained with the polarographic oxygen sensor in the TIMR method were compared with close-system measurements made using an optical oxygen sensor (Loligo Systems, Copenhagen, Denmark) connected to a PC-controlled fiber optic trace oxygen transmitter (Witrox 4) in 23 independent fish acclimated to 28 and 32°C, and exposed to TIMR min (Figure 3A) and TIMR max (Figure 3B) (5 or 6 fish for each TIMR evaluation and experimental temperature). The results confirmed that the oxygen sensors used led to similar results (Figure 3C). In the clownfish experiments, intermittent respirometry chambers combined with optode oxygen sensors were used to measure oxygen consumption (Díaz et al., 2007) (Loligo Systems, Viborg, Denmark).

### Lactate measurements

After each swim-flume trial and analysis of HMR obtained in TIMR max or chase protocol measurements, fish were gently placed in a seawater tank with clove oil (100 mg L^−1^) to be anesthetized before blood samples were taken for lactate measurement. Fish blood was obtained using a pre-cooled and pre-heparinized disposable insulin syringe with a 30 G needle from the caudal vein using the lateral approach. Lactate concentration was immediately measured on a disposable lactate test strip (Lactate Plus Meter, Nova Biomedical, Waltham, MA, USA). Data recorded with the Lactate Plus Meter were validated through a standard curve (*n* = 3) of simultaneous measurements using a commercial kit (Trinity Biotech). As no significant differences were detected between methods (*T*-test; *p* > 0.05), only the Lactate Plus Meter was subsequently used to evaluate the lactate concentration. In clownfish, blood lactate levels were not monitored due to the small size of the specimens.

### Data analysis and representation

In the TIMR method, thermal metabolic scope (TMS) was defined as:

(4) $$\begin{array}{r} TMS=HMR-LMR \end{array}$$

where HMR = high metabolic rate and LMR = low metabolic rate (both as mg O_2_ h^−1^ g WW^−1^).

Values of temperature coefficient Q_10_ were calculated as:

(5) $$\begin{array}{r} Q_{10}=\;{\left(\frac{\left(\text{HMR}\right)}{\left(\text{LMR}\right)\;}\right)}^{\frac{10}{TIMR\;max\;-TIMR\;min}} \end{array}$$

where HMR = high metabolic rate, LMR = low metabolic rate (both as mg O_2_ h^−1^ g WW^−1^), TIMR max = temperature at which HMR was measured, and TIMR min = temperature at which LMR was measured. Those temperatures were calculated from the critical temperature (CT max and min) at each acclimation temperature detailed in Table 1.

Using the chase method, aerobic scope (AS) was defined as:

(6) $$\begin{array}{r} AS=Chase\;MMR-RMR \end{array}$$

where chase MMR = maximum metabolic rate obtained with the chase method and RMR = lowest values of routine metabolic rate (both expressed as mg O_2_ h^−1^ g WW^−1^) over 24 h following the protocol proposed by Chabot et al. (2016b).

All figures and statistical analyses were done using R (R Core Team, 2017). For both experiments, the effect of acclimation temperature was evaluated using a one-way ANOVA at *p* < 0.05, with each method analyzed separately (swim-flume, chase, and TIMR method) to evaluate the effects of acclimation temperature on fish metabolism. When ANOVA detected statistical differences, a Tukey HSD test was used to evaluate possible differences between fish metabolic mean values obtained in different acclimation temperatures. This procedure allowed to detect the acclimation temperature at which changes in the metabolic rate occurred using each of the three methods independently.

## Results

### Swim-flume and TIMR test comparison

**Figure 4:**
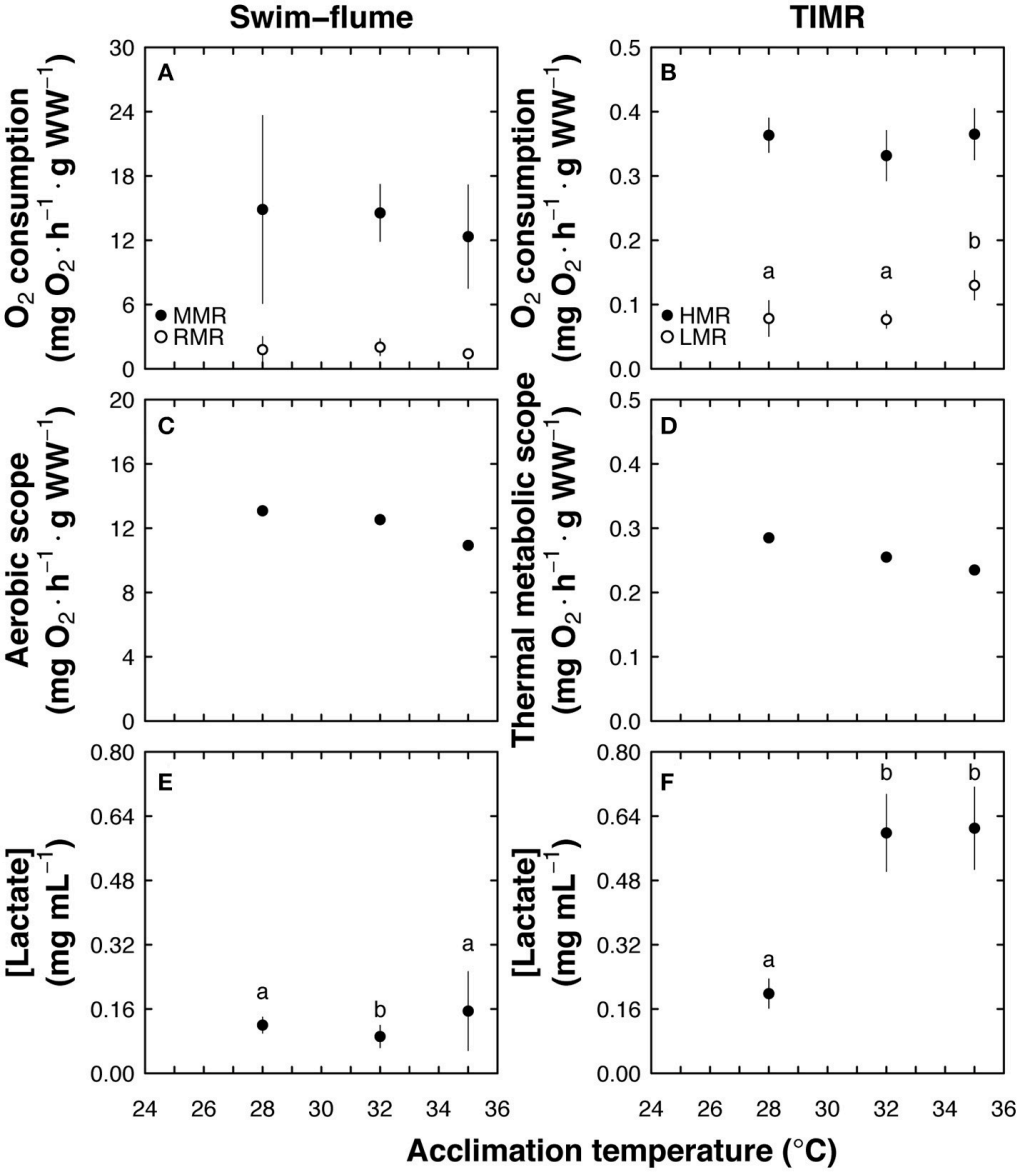
Temperature-induced metabolic rate (TIMR) method comparison with swim-flume respirometry using *Centropomus undecimalis* acclimated to 28 (optimal), 32, and 35°C (sub-optimal) temperatures. **(A)** Maximum (MMR; mg O_2_ h^−1^ g^−1^ WW; black circles) and routine metabolic rates (RMR; mg O_2_ h^−1^ g^−1^ WW; white circles) obtained in the swim-flume respirometer at 2 and 9 cm s^−1^, respectively, **(B)** high and low metabolic rates (HMR and LMR; mg O_2_ h^−1^ g^−1^ WW; black and white circles, respectively) obtained at TIMR max (90% of CT max) and min (110% of CT min), **(C)** aerobic scope (AS = mean MMR—mean RMR; mg O_2_ h^−1^ g^−1^ WW; swim-flume respirometry), and **(D)** thermal metabolic scope (TMS = mean HMR—mean LMR; mg O_2_ h^−1^ g^−1^ WW; TIMR method; black circles). Blood lactate concentration (mg mL^−1^) were measured after **(E)** MMR (swim-flume respirometry) and **(F)** HMR (TIMR method) assays; MMR, RMR, HMR, LMR, and blood lactate concentration data are graphed as mean ± SD; *n* = 6 at each acclimation temperature and for each method; different letters indicate statistical differences between acclimation temperatures at *p* < 0.05.

The MMR of fish measured in the swim-flume respirometer did not change significantly with acclimation temperature, and was 20.4, 7.5, and 10.5-fold higher than corresponding RMR values for treatments at 28, 32, and 35°C, respectively (Figure 4A). Low metabolic rates (LMR) measured using the TIMR method were affected by acclimation temperature, with significantly lower values in animals acclimated to 28 and 32°C, and higher in animals acclimated to 35°C (*p* < 0.001; Figure 4B). In contrast, there were no statistical differences between treatments in animals exposed to TIMR max (*p* > 0.05; Figure 4B). High metabolic rate (HMR, measured at TIMR max) was 4.6, 4.3 and 2.8-fold higher than LMR obtained in animals acclimated to 28, 32 and 35°C, respectively (Figure 4B). Aerobic scope (AS) and thermal aerobic scope (TMS) values showed an inverse relationship with temperature, with high values in fish acclimated to 28°C and lower in fish acclimated to 35°C (Figures 4C,D). Therefore, independent of the method used, a similar effect of temperature was observed in the relationship between AS, TMS, and temperature.

Lactate values tested after maximum activity in the swim-flume respirometer fluctuated between 0.12 and 0.15 mg ml^−1^, with lower values registered in animals acclimated to 32°C than those obtained in fish acclimated to 28 and 35°C (*p* < 0.01; Figure 4E). Lactate values of fish tested with the TIMR method were affected by the higher acclimation temperatures, with lower values (0.19 mg ml^−1^) in animals acclimated to 28°C and higher values in animals acclimated to 32 and 35°C (mean value of 0.6 mg ml^−1^; *p* < 0.008, Figure 4F).

### Chase and TIMR method comparison

**Figure 5:**
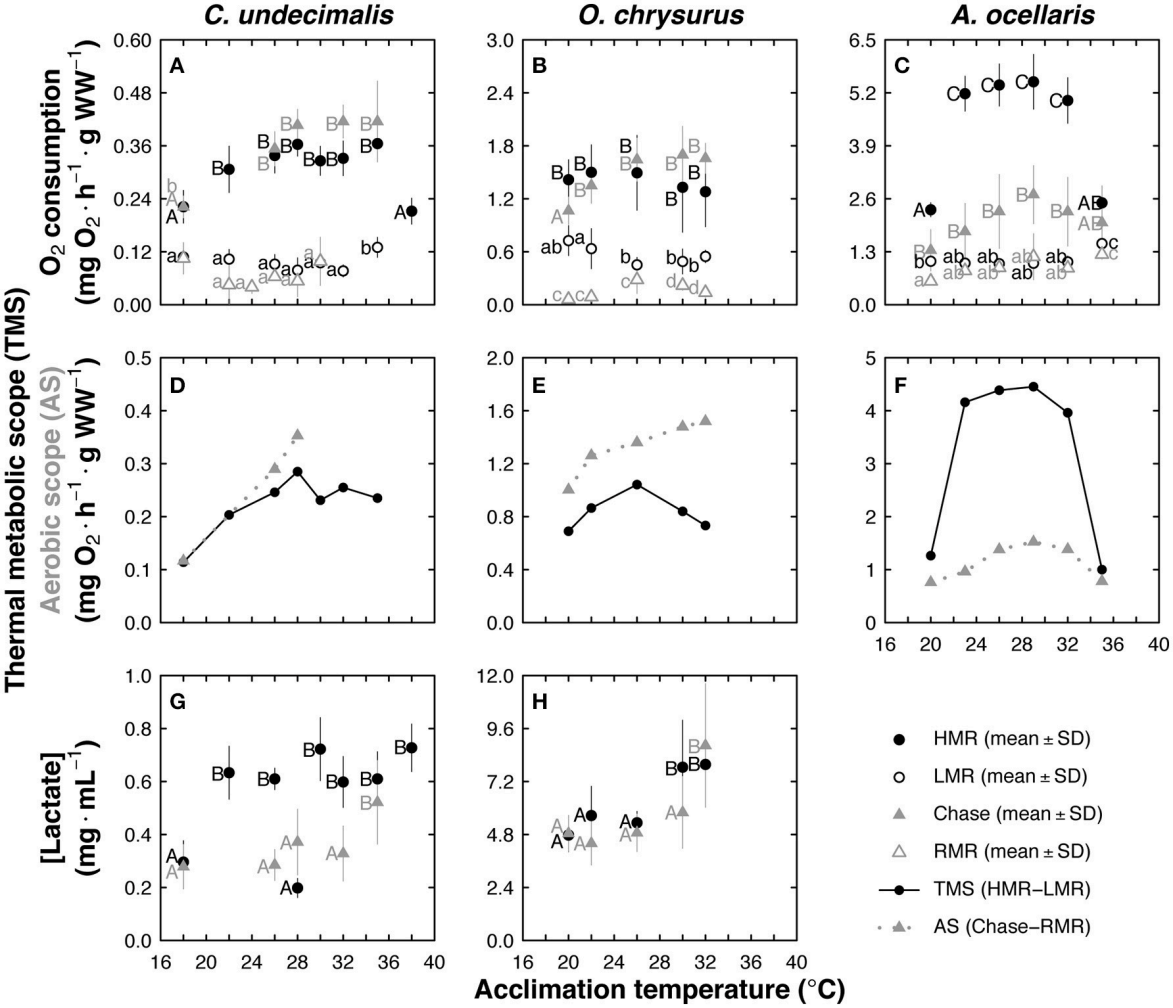
Effect of acclimation temperature in *Centropomus undecimalis* (Cu), *Ocyurus chrysurus* (Oc), and *Amphiprion ocellaris* (Ao), using chase and temperature-induced metabolic rate (TIMR) methods. Metabolic rates obtained from the chase method (mg O_2_ h^−1^ g^−1^ WW; gray filled triangles), routine metabolic rate (RMR) from 24 h respiration measurement (mg O_2_ h^−1^ g^−1^ WW; gray empty triangles), high and low metabolic rates (HMR and LMR; mg O_2_ h^−1^ g^−1^ WW; black and white circles, respectively) obtained at TIMR max (90–95% of CT max) and min (105–110% of CT min) are shown for **(A)** Cu, **(B)** Oc, and **(C)** Ao. Aerobic scope (AS mg O_2_ h^−1^ g^−1^ WW; gray line) and thermal metabolic scope (TMS; mg O_2_ h^−1^ g^−1^ WW; black line) were calculated for **(D)** Cu, **(E)** Oc, and **(F)** Ao. Blood lactate concentration (mg mL^−1^) were measured after chase and HMR for **(G)** Cu and **(H)** Oc. Chase, RMR, HMR, LMR, and blood lactate concentration data are graphed as mean ± SD; *n* = 6 at each acclimation temperature and for each method; different letters indicate statistical differences between acclimation temperatures at *p* < 0.05 obtained separately within each method. Upper and lower case correspond to high and low metabolic rates, respectively.

*Centropomus undecimalis* LMR values were constant in animals acclimated to temperatures between 18 and 32°C but increased at 35°C (*p* < 0.008; Figure 5A). Lower HMR values were recorded in snook acclimated to 18 and 38°C (*p* < 0.001; 0.22 mg O_2_ h^−1^ g WW^−1^) compared with those obtained with other acclimation treatments (0.34 mg O_2_ h^−1^ g WW^−1^; Figure 5A). No significant statistical differences were found between HMR (TIMR method) and MMR (chase method) and between LMR (TIMR method) and RMR (chase method) in snook (*p* > 0.05; Figure 5A).

*Ocyurus chrysurus* LMR was lower in fish acclimated from 26 to 32°C than observed in those acclimated to 22°C (*p* < 0.01; Figure 5B). Also, LMR (TIMR method) values were higher than RMR (used to calculate AS with the chase method) in fish acclimated at all experimental temperatures (*p* < 0.001; Figure 5B). When maximum metabolic rates were compared, HMR (TIMR method) and MMR (chase method) were similar along acclimation temperatures (*p* > 0.05; Figure 5B). Two-way ANOVA showed that there were no significant differences between metabolic rates (MMR and HMR) in fish acclimated at 22 to 32°C (mean value of 1.55 mg O_2_ h^−1^ g WW^−1^; *p* > 0.05, Figure 5B). In contrast, lower values were registered in chased fish acclimated to 20°C than obtained in fish metabolic rate measured with TIMR method (*p* < 0.008; Figure 5B).

*Amphiprion ocellaris* (clownfish) LMR and RMR values were constant along all acclimation temperatures, with a mean value of 1.11 ± 0.1 mg O_2_ h^−1^ g WW^−1^ (*p* > 0,05; Figure 5C). In this species, HMR (TIMR method) were between 123 and 288% higher than MMR (chase method), with the most pronounced differences in fish acclimated between 22 and 32°C (*p* < 0.001; Figure 5C). Both HMR and MMR remained constant in the thermal range of 22–32°C, with mean values of 5.24 (HMR) and 2.6 (MMR, chase) mg O_2_ h^−1^ g WW^−1^ (Figure 5C). A significantly lower metabolic rate was observed in clownfish acclimated to 20 and 35°C using the TIMR and chase methods, with no significant differences between methods at these extreme temperatures (*p* > 0.05; Figure 5C).

Snook TMS (TIMR method) and AS (chase method) showed that highest performance was between 28 and 32°C acclimation temperatures and at 28°C, respectively (Figure 5D). It is worth noting that this fish species was more sensitive to low temperatures than high temperatures (Figure 5D). Values of AS resulted slightly higher than values obtained when calculated TMS (Figure 5D). In yellow tail fish, a TMS spike was observed in animals acclimated to 26°C, and no decreasing pattern was seen with chase method at 30 and 32°C (Figure 5E).

Although the HMR (TIMR method) and MMR (chase method) were significantly different in clownfish, those methods produced similar curves, with high metabolic performance at acclimation temperatures between 22 and 32°C (Figure 5F). This species was highly affected by acclimation temperatures lower than 22°C or higher than 32°C, with TMS significantly reduced at 35°C (Figure 5F). Values of Q_10_ of snook, yellow tail and clownfish showed that during TIMR method evaluation, temperature changed the fish metabolism producing values between 1.3 and 3.1. When Q_10_ values were related with acclimation temperatures a bell-shaped curve was observed with maximum values of Q_10_ in animals acclimated from 22 to 32°C for all species (Figure S1).

Snook blood lactate levels measured after activity (TIMR or chase method) were shaped differently by acclimation temperature depending on the method used; higher values were registered using the TIMR method than the chase method (*p* < 0.001; Figure 5G), except at 28°C. After chasing, there were no significant differences in lactate levels of snook acclimated between 18 and 32°C (*p* > 0.05; mean value of 0.31 ± 0.04 mg ml^−1^; Figure 5G). However, in animals acclimated to 35°C blood lactate levels were significantly higher after the chase protocol (0.52 ± 0.16 mg ml^−1^; *p* < 0.001; Figure 5G). After the HMR measurement, fish acclimated to 22, 26, 30, 32, 35 and 38°C showed similar lactate levels with a mean value of 0.65 ± 0.06 mg ml^−1^ (*p* > 0.05; Figure 5G), while fish acclimated to 18 and 28°C showed a mean lactate value 62% lower (0.26 ± 0.07 mg ml^−1^) than the other treatments (Figure 5G). In yellow tail fish, similar values were obtained with both methods in animals acclimated from 20 to 26°C, with a mean value of 5.08 ± 0.3 mg ml^−1^ (*p* > 0.05; Figure 5H). Higher lactate levels were observed following the TIMR method at and above 28°C, while the same magnitude was reached at 32°C for the chase protocol (*p* < 0.001; Figure 5H).

## Discussion

The present new approach (TIMR method) showed that TMS, without being a direct measurement of AS, can be useful to identify how acclimation temperature modulates metabolic performance in non-athletic fish. This method opens the door to evaluate the aerobic power budget of aquatic species that are immobile or display very little movement, namely sedentary invertebrates and non-athletic fish. Using the TIMR method, animals are exposed at two different temperatures; one provoking low and other high metabolic rates, whose difference (= TMS) allowed the assessment of its metabolic capabilities. In the three-fish species, the TIMR method consistently resulted in an increment of metabolic rate (TMS = HMR–LMR), within physiologically expected ranges (Q_10_ > 1 and ≈ 3; Table S2) indicating that 5 min exposure to low (5–10% higher than CT min) and high (5–10% lower than CT max) temperatures is enough to obtain a metabolic response that reflects the aerobic power budget of aquatic species. Long-term thermal acclimation changes the metabolic capacities of animal tissues. Accordingly, in long-term acclimated animals, the short-term analysis of TMS was useful to evaluate the consequences of thermal acclimation for the aerobic power budget.

The TIMR method shows its applicability when it is compared to conventional swim-flume and chase protocols. The same phenomena were reported supporting the same conclusions for the effects of temperature on metabolic performance of the athletic fish species *Centropomus undecimalis*. In the TIMR *vs*. swim-flume comparison, fish acclimated to optimal and sub-optimal temperatures (Noyola Regil et al., 2015) tended to have reduced TMS and AS at increased temperatures. In fact, an increase of blood lactate was registered in temperatures higher than 28°C, indicating that with both methods it is possible to see changes in other components of fish metabolism. The TIMR method was also shown to be useful for the study of fish species with different swimming behaviors. In semi-sedentary fish (yellow tail) or well-defined sedentary fish (clownfish), similar or higher metabolic responses were found with the TIMR method than the chase method. In clownfish, a stronger stimulus was needed to reveal its aerobic power budget, and the results were stronger with the TIMR method than with chasing. It is not surprising that this sedentary species showed higher metabolic rates after temperature stimulation than after chasing (Madeira et al., 2016a; Habary et al., 2017). This species is particularly reluctant to move, and consistently attempts to reduce its activity in spite of the mechanical stimulus exerted during chasing.

We presented evidence showing that relatively high and low temperatures can induce maximum and minimum metabolic activity, not only through muscle activity as in traditional methods (Fry, 1947; Brett, 1964; Norin and Clark, 2016), but through the stimulating effects of temperature on respiration (see Table S2). The TIMR method may be considered a simple, fast, and standardized method to accurately evaluate metabolic scope from effects of temperature on metabolic rate, as compared to the conventional methods used to evaluate SMR and MMR. Although this method requires the previous evaluation of CT max and min, nowadays there are many available literature data of those limits that can be used to calculate the TIMR max or min (as 90–95% and 5–10% CT max and min, respectively) and test the method in invertebrates (crustaceans and mollusc) and fish (For additional data on CT max and min see Bennett et al., 2018). A table with 121 data of thermal limits of aquatic ectotherms has been included (see Table S1). Otherwise, there are many invertebrate species of ecological and aquaculture interest like shrimps, crabs, crayfish etc., with which the conventional methods of evaluation of AS cannot be used, because their metabolic scope cannot be evaluated in a swim-flume or by pinching or chasing. In this sense the TIMR method can be considered an alternative to AS evaluations.

Taking into consideration that the TIMR method reflects how temperature stimulates the biochemical reactions of ectotherms (Fry, 1971), while the swim-flume involves visibly high muscular demands, the same magnitude of metabolic rates cannot be expected from the two methods. Despite these differences, similar curves that relate temperature and metabolism were obtained, indicating that the TIMR method can be used to evaluate the effects of temperature on the metabolic performance of those organisms that cannot be studied using the swim-flume method, such as sedentary ectotherms or sluggish fish. In a recent study the TIMR method was used to evaluate the thermal limits of juveniles of the lobster *Panulirus argus* (Rodríguez-Fuentes et al., 2017). In that study, the advantages of the TIMR method were demonstrated by obtaining the maximum metabolic performance of lobsters acclimated to different temperatures. Although in the same study, it was also showed that TMS couldn't be used as the sole indicator to define the optimal thermal range of this species. Indeed, when complemented with growth rates and other physiological indices (oxidative stress indicators and metabolites), TMS data were key in defining the thermal optimal and sub optimal (*pejus*) thermal limits of lobster (Rodríguez-Fuentes et al., 2017).

Chase and TIMR methods work in different ways. In the TIMR method the energy power budget observed was, in the first place, a result of temperature effects on energetic mechanisms at mitochondrial level that followed the energetic demands provoked by temperature (Q_10_, Table S2). Further studies should be carried out in an attempt to determine in more detail how the TIMR method affects the mitochondrial machinery, similar to what was done when the metabolic processes were evaluated at the critical swimming speed, as part of the AS method (Lurman et al., 2007). In animals exposed to the chase method, MMR corresponds to the sum of the fish's metabolic rate expressed at a particular acclimation temperature plus the increment of metabolic rate provoked only by muscular activity. In this method, besides the effect of acclimation temperature, metabolic stimulation is reflected in the excess post-exercise oxygen consumption (EPOC) which might include physiological debts (as lactate, reactive oxygen species and other molecules) that influence metabolic rate (Norin and Clark, 2016).

One of the challenges in AS studies is the evaluation of SMR. According to Chabot et al. (2016b) SMR can be defined “… as the rate of energy expenditure of a fish that is in post-absorptive, calm, inactive state after proper thermal acclimation.” In the TIMR method, when evaluating LMR in 21 days acclimated fish, the oxygen consumption is obtained in a forced inactive state that is enough to cause a low metabolic rate, but not enough to provoke any other physiological effect. During LMR, it is possible to hypothesize that the metabolism is slower due to the decrease in the activity and cost of transmembrane ion exchange covered by the enzymes involved in the electron transport chain in the mitochondria, which leads to a lower energy production and a reduction of the general activity of the fish in the respirometric chamber. So, LMR, without being strictly equivalent to SMR, allows calculating a low and standardized routine oxygen consumption, and obtain reliable metabolic data to assess the TMS. In this sense, the present study demonstrated that in three fish species, LMR followed the same behavior of RMR. This indicates that, regardless of the metabolic level reached, LMR as a part of TMS is useful to evaluate the effects of temperature, because as was mentioned early, the relationship between TMS and temperature produced similar curves than obtained when a curve of AS and temperature was constructed (Figure 1).

Many of the interpretations related to AS potential for thermal performance evaluation depend on accurate measurements of metabolic rates (Ern et al., 2014, 2015; Norin et al., 2014; Svendsen et al., 2016). To carry out those measurements, researchers have traditionally used polarographic (Ern et al., 2014, 2015) and, more recently optode sensors (Norin et al., 2014). In the present study, we used both types of sensors in an attempt to demonstrate that either could be used if correct practices are followed (Svendsen et al., 2016). Results obtained upon comparison of the handheld and optical sensors on the oxygen consumption measurements of snook indicate that there were no statistical differences between methods, validating the use of those sensors in fish. Other studies have obtained AS of invertebrates using polarographic sensors and the chase protocol. Ern et al. (2014) used a Hamilton type sensor previously calibrated at different temperatures to assess the effects of temperature on the SMR and MMR of *Macrobrachium rosenbergii*. Those authors stabilized the sensor by maintaining it for 1 h in the new temperature before measurements. A similar protocol was followed in the present study when the polarographic sensor was used allowing fast and reliable oxygen measurements as in the Ern et al. (2014) study.

Blood lactate is a component of anaerobic metabolism, and it increases during intense exercise. Lactate levels together with cardiorespiratory evaluations have been used to define when animals exposed to MMR switch from aerobic to anaerobic metabolism (Santos and Keller, 1993; Clark et al., 2011; Healy and Schulte, 2012; Giomi and Pörtner, 2013; Rodríguez-Fuentes et al., 2017). Although further studies are needed to define at exactly what point blood lactate levels indicate critical increases in anaerobic metabolism, results obtained in the present study demonstrate that when the TMS was reduced, lactate levels increased. This suggests that lactate was released to blood, presumably as anaerobic metabolism increased. To better understand the relationship between TMS and lactate metabolism, other elements of the anaerobic biochemical pathway and probably other components such as the redox system should be evaluated in these fish species. A recent study found high lactate mobilization in lobsters acclimated to 30°C that coincided with a reduction in growth rate, TMS and high activity of superoxide dismutase and Glutathione-s-transferase enzymes of the redox system. This information was used to more precisely define the optimal thermal limits and amount of hemolymph lactate that can be considered as an indicator of anaerobic metabolism in *P. argus* (Rodríguez-Fuentes et al., 2017).

Thus, the TIMR method provides an alternative way to estimate the difference between high and low metabolic activity under different acclimation conditions that, although not conceptually equivalent to aerobic scope, may allow the standardized estimation of TMS that is particularly relevant for sedentary species where measurement of aerobic scope via maximal swimming is inappropriate.

### Advantages of the TIMR method

Results herein show that the TIMR method produces temperature-metabolic rate relationships that are comparable to those obtained with methods used in classical fish physiology. Different magnitudes of oxygen consumption values were noted when the TIMR method was compared with the swim-flume method, but results were similar when compared to the chasing method. The temperature used for the TIMR max measurement causes a direct rise of oxygen consumption thanks to the effect that high temperatures have on the enzymes involved in energy production at the mitochondrial level. In contrast, constant swimming activity (swim-flume and chasing of athletic fish) increases the metabolic rate through increased muscular energy demand, which in turn increases oxygen consumption. Despite those differences, both methods obtain similar tendencies of AS and TMS when temperature was tested as a factor of acclimation. Both mechanical stimulus (chase) and temperature (TIMR) methods allow the evaluation of metabolic performance which, for purposes of standardization could be denominated in both cases as a method for the evaluation of the “scope for metabolic activity” as introduced by Fry (1947).

What sets this method apart from the conventional methods is that TIMR is suitable for studying the effects of temperature on the metabolic capacities of well-defined sedentary aquatic organisms such as clownfish (unpublished data), lobster (Rodríguez-Fuentes et al., 2017) or other non-mobile organisms and even embryos (Ide et al., 2017). As with other methods, TIMR should be tested on other key marine sedentary organisms, such as mollusc, sea urchins, and worms (among others) to define the limits in the application of this novel method. Through such testing, TIMR may prove to be a useful tool for monitoring physiological changes associated with temperature, and possibly with other environmental factors that vary naturally in aquatic ecosystems, such as pH, dissolved oxygen or pollutants.

### Disadvantages of the TIMR method

The principal disadvantage of this method is that prior to use, the researcher is required to evaluate critical thermal maximum (CT max) and critical thermal minimum (CT min), observing the behavior of animals to identify the relationship between temperature, time of exposure and escape behavior. However, CTmax and min is only a thermal reference where a researcher can identify the high temperature that enhances the metabolic rate of organisms. While the use of CTmax and min provide a basis for standardization criteria of thermal conditions for the evaluation of TMS, the researcher, to evaluate TMS, could also choose a high and low non-lethal temperature that provoke a higher and lower metabolic rate than observed in animals that are maintained in routine conditions. In this form the disadvantage related with the evaluation of CTmax or min can be minimized. In this case, is recommended to the researcher justify widely the high and low temperatures used to evaluate TMS.

The critical thermal method (CT max and CT min) has been clearly used for many years, giving interesting and useful information on thermal biology of many temperate and tropical species (Reynolds and Casterlin, 1979; Díaz et al., 2015; Madeira et al., 2016a,b; Vinagre et al., 2016; Habary et al., 2017), and providing a strong foundation for its use. In this sense a table with many published data on CT max and CT min was provided in an attempt to offer referenced values to calculate TIMR max and min and evaluate TMS (Table S1). Also, more than 2000 data on CT max and min were recently published helping researchers to obtain easily the data to calculate TIMR max or min temperatures. Depending on the thermal sensitivity of each species, it is possible that the proportion of CT max and CT min that should be used in TIMR evaluations could change, as observed in the present study, changing between 90 and 95% of CT max or 110 or 105% of CT min. The last value to be used will depend on the accuracy of the critical temperatures evaluations and sensitivity of the organism under study. Also, researchers should consider that TIMR max and min are proposed to obtain only an HMR (not necessarily the maximum one), and LMR (not necessarily the basal metabolism), but standardized high or low metabolic rate.

## Ethics statement

Animal ethics approval was not required for the use of cultured fish in research in Mexico, in accordance with national guidelines. However, for all experiments and animal husbandry, we used established criteria for good management practices for aquatic animals based on the norms of conduct of National Autonomous University of Mexico and Centro de Investigación Científica y de Educación Superior de Ensenada.

## Author contributions

KP and PG provided the idea of TIMR, designed the method, and contributed to the writing of the manuscript. JA conducted experiments, analyzed the corresponding data, and contributed to the writing of the manuscript as the first author of the paper. MM contributed to data analysis and revision of the manuscript. EL-R conducted lactate evaluations of blood. FD and DR conducted the TIMR experiments and contributed to the writing of the manuscript. CC-M conducted experiments and contributed to the general discussion of results. NT conducted the experiments, analyzed the results, draw the figures, and contributed to writing the manuscript. H-OP contributed to data analysis and revision of the manuscript. CR contributed to the method design, conducted experiments, analyzed the data, and wrote the manuscript.

### Conflict of interest statement

The authors declare that the research was conducted in the absence of any commercial or financial relationships that could be construed as a potential conflict of interest.

## Supplementary material

Table S1 Critical thermal maxima and minima of several species of mollusc (m), crustacean (c), and fish (f) groups, with respective geographic coordinates and habitat (sw= seawater; fw= freshwater; and t= terrestrial).

Table S2 Values obtained from LMR and HMR measured at TIMR min and TIMR max conditions for 5 min for Centropomus undecimalis (Snook), Ocyurus chrysurus (Yellow tail) and Amphyprion ocellaris (clownfish).

Figure S1 Effect of acclimation temperature in Q_10_ values obtained using data of oxygen consumption (Low and high metabolic rates, i.e., LMR and HMR) of the three fish species exposed at the temperatures used to induce metabolic rates (TIMR min and max, respectively) at each acclimation temperature. Note that data of clownfish where placed in the secondary axis to improve its visualization.

Supplemental Video 1 *Centropomus undecimalis* in the acrylic swim-flume respirometry chamber maintained at a water speed of 2 cm s^−1^.

Supplemental Video 2 The chase protocol applied to *Centropomus undecimalis*.

Supplemental Video 3 *Centropomus undecimalis* in the closed respirometry chamber after chase protocol.

Supplemental Video 4 *Ocyurus chrysurus* juvenile during TIMR min measurement in a closed circulation setting.

Supplemental Video 5 *Ocyurus chrysurus* juvenile during TIMR max measurement in a closed circulation setting.
