# Supplementary material for: Comparison of Aerobic Scope for Metabolic Activity in Aquatic Ectotherms With Temperature Related Metabolic Stimulation: A Novel Approach for Aerobic Power Budget
Source: Front Physiol. 2018 Oct 22;9:1438. doi: 10.3389/fphys.2018.01438 (PMC6204536; doi:10.3389/fphys.2018.01438)
Supplement: Table S1 — Critical thermal maxima and minima of several species of mollusc (m), crustacean (c), and fish (f) groups, with respective geographic coordinates and habitat (sw= seawater; fw= freshwater; and t= terrestrial). [file Table_1.DOCX]

| **sT1.**Critical thermal maxima and minima of several species of mollusc (m), crustacean (c), and fish (f) groups, with respective geographic coordinates and habitat (sw= seawater; fw= freshwater; and t= terrestrial) | | | | | | | | | | | | | |
| --- | --- | --- | --- | --- | --- | --- | --- | --- | --- | --- | --- | --- | --- |
|  |  |  |  |  |  |  | **CT min** | | | **CT max** | | |  |
| **Genus** | **species** | **lat** | **lon** | **habitat** | **group** | **Accli. (d)** | **rate (°C/min)** | **min** | **mean** | **rate (°C/min)** | **max** | **mean** | **Reference** |
| *Aplysia* | *californica* | 32.25 | 116.98 | sw | m | 21 | NA | NA | NA | 0.06 | 36.2 | 0.58 | Re et al. 2013 |
| *Arbacia* | *incisa* | 31.43 | -116.45 | sw | m | 42 | NA | NA |  | 1 | 38.4 | 0.213 | Diaz et al. 2017 |
| *Conasperella* | *perplexus* | 33.79 | -118.30 | sw | m | 15 | NA | NA | NA | 1 | 40 | NA | Lugo et al. 2016 |
| *Conasperella* | *ximenes* | 30.54 | -114.41 | sw | m | 15 | NA | NA | NA | 1 | 39.5 | NA | Lugo et al. 2016 |
| *Californiconus* | *californicus* | 32.34 | -117.06 | sw | m | 15 | NA | NA | NA | 1 | 31 | NA | Lugo et al. 2016 |
| *Haliotis* | *fulgens* | 31.27 | -116.37 | sw | m | 28 | NA | NA | NA | 0.3 | 33.6 | NA | Diaz et al. 2006 |
| *Haliotis* | *corrugatta* | 31.27 | -116.37 | sw | m | 28 | NA | NA | NA | 0.3 | 32 | NA | Diaz et al. 2006 |
| *Haliotis* | *rufescens* | 31.85 | -116.61 | sw | m | 8 | NA | NA | NA | 0.3 | 27.5 | NA | Diaz et al. 2000 |
| *Kelletia* | *kelleti* | 31.42 | -116.45 | sw | m | 21 | 0.1 | 9.5 | 0.14 | 0.1 | 30.3 | 0.16 | Re et al. unpublished |
| *Megastrea* | *lithopoma undosa* | 31.42 | -116.45 | sw | m | 21 | NA | NA | NA | 0.03 | 29.7 | NA | Diaz et al. 2011 |
| *Megatura* | *crenulata* | 31.42 | -116.45 | sw | m | 21 | NA | NA | NA | 0.3 | 28.8 | 0.18 | Diaz et al. 2015 |
| *Melongena* | *corona vispinoza* | 21.18 | -90.03 | sw | m | 21 | 0.2 | 7.6 | 0.34 | 0.2 | 42.1 | 0.23 | Noyola et al. 2015 |
| *Octopus* | *maya* | 21.18 | -90.03 | sw | m | 20 | 1 | 11.6 | 0.81 | 1 | 36.5 | 0.32 | Noyola et al. 2013 |
| *Octopus* | *mimus* | 23.30 | -70.25 | sw | m | 4 | 0.68 | 9 | NA | 1.21 | 33.3 | NA | Zuñiga et al. 2012 |
| *Tegula* | *regina* | 31.42 | -116.45 | sw | m | 21 | 0.06 | NA | NA | NA | 31.2 | 0.32 | Salas et al. 2014 |
| *Asellus* | *aquaticus* | 60.45 | 22.27 | fw | c | 15 | NA | NA | 7.957 | 0.2 | 34.9 | 0.58 | Lagerspetz and Bowler 1993 |
| *Callinectes* | *similis* | 21.18 | -90.03 | sw | c | 21 | 0.3 | 10.35 |  | 0.3 | 41.5 | 0.25 | Noyola et al. 2015 |
| *Cancer* | *antennarius* | 31.27 | -116.37 | sw | c | 21 | NA | NA | NA | 0.5 | 32.7 | 0.1-0.3 | Padilla et al. 2012 |
| *Cancer* | *pagurus* | 54.69 | -1.16 | sw | c | 21 | NA | NA | NA | 0.2 | 30.6 | 0.49 | Cuculescu et al. 1998 |
| *Carcinus* | *maenas* | 54.69 | -1.16 | sw | c | 21 | NA | NA | NA | 0.2 | 35.8 | 0.16 | Cuculescu et al. 1998 |
| *Cherax* | *quadricarinatus* | *18.62* | *-99.22* | fw | c | 30 | NA | NA | NA | 1 | 42 | 0.47 | Diaz et al. 2004 |
| *Crangon* | *crangon* | 50.34 | -4.15 | sw | c | 14 | 0.2 | -1.1 | 0.28 | 0.75 | 35.4 | 0.2 | Reiser 2013 |
| *Farfantepenaeus* | *aztecus* | 21.61 | -97.55 | sw | c | 21 | NA | NA | NA | 1 | 42 | 0.5 | Re et al. 2005 |
| *Hemigrapsus* | *nudus* | 48.90 | -125.23 | sw | c | 14 | 0.5 | 3.5 | 0.22 | 0.5 | 33.6 | 0.42 | McGaw 2003 |
| *Hemigrapsus* | *crenulatus* | 41.37 | -73.35 | sw | c | 21 | 1 | 3.2 | 0.275 | 0.5 | 34.9 | 0.272 | Cumillaf et al. 2016 |
| *Homarus* | *americanus* | 41.90 | NA | sw | c | 21 | NA | NA | NA | 0.75 | 30 | 0.31 | Camacho et al. 2006 |
| *Libinia* | *dubia* | 21.18 | -90.03 | sw | c | 21 | 0.3 | 9.4 | 0.54 | 0.3 | 41.5 | 0.55 | Noyola et al. 2015 |
| *Litopenaeus* | *vannamei* | 12.17 | NA | sw | c | 30 | 0.02 | 7.5 | 0.24 | 0.02 | 42.2 | 0.41 | Kumlu et al. 2010 |
| *Macrobrachium* | *tenellum* | 26.90 | -112.00 | fw | c | 21 | NA | NA | NA | 1 | 43 | 0.58 | Hernandez-Rodriguez et al. 1996 |
| *Macrobrachium* | *acanthurus* | 18.37 | -92.87 | fw | c | 30 | 1 | 11 | 0.44 | 1 | 39.8 | 0.48 | Diaz et al. 2002 |
| *Macrobrachium* | *malcolmsonii* | 11.48 | 79.77 | fw | c | 30 | NA | NA | NA | 0.3 | 41.4 | 0.37 | Selvakumar and Geraldine 2005 |
| *Macrobrachium* | *rosenbergii* | 3.96 | NA | sw | c | 28 | 1 | 10.5 | 0.5 | 1 | 42 | 0.47 | Diaz et al. 1998 |
| *Orchestia* | *gammarellus* | 65.92 | -22.43 | t | c | 10 | NA | NA | NA | 1 | 38.6 | 0.14 | Morritt and Ingolfsson 2000 |
| *Orchestia* | *gammarellus* | 65.82 | -22.49 | t | c | 10 | NA | NA | NA | 1 | 37.9 | 0.15 | Morritt and Ingolfsson 2000 |
| *Orchestia* | *gammarellus* | 63.80 | -22.72 | t | c | 10 | NA | NA | NA | 1 | 37.7 | 0.17 | Morritt and Ingolfsson 2000 |
| *Orchestia* | *gammarellus* | 64.02 | -22.16 | t | c | 10 | NA | NA | NA | 1 | 37.9 | 0.19 | Morritt and Ingolfsson 2000 |
| *Oronectes* | *rusticus* | 39.44 | -84.52 | fw | c | 6 | NA | NA | NA | 0.6 | 41.3 | 0.24 | Claussen 1980 |
| *Oronectes* | *virilis* | 39.44 | -84.52 | fw | c | 6 | NA | NA | NA | 0.6 | 39.2 | 0.18 | Claussen 1980 |
| *Palaemon* | *elegans* | 50.36 | -4.13 | sw | c | 7 | NA | NA | NA | 0.75 | 35 | 0.3 | Magozzi and Calosi 2015 |
| *Palaemon* | *serratus* | 50.35 | -4.13 | sw | c | 7 | NA | NA | NA | 0.75 | 33.5 | 0.31 | Magozzi and Calosi 2015 |
| *Palaemon* | *varians* | 50.72 | -1.52 | sw | c | 7 | NA | NA | NA | 0.75 | 36 | 0.23 | Magozzi and Calosi 2015 |
| *Palaemon* | *montagui* | 51.49 | 0.85 | sw | c | 7 | NA | NA | NA | 0.75 | 27.6 | 0.26 | Magozzi and Calosi 2015 |
| *Palaemon* | *macrodactylus* | 28.16 | NA | sw | c | 7 | NA | NA | NA | 0.75 | 37.8 | 0.39 | Magozzi and Calosi 2015 |
| *Palaemonetes* | *varians* | 48.66 | -1.62 | sw | c | 120 | NA | NA | NA | 0.93 | 35.9 | 0.5 | Ravaux et al. 2012 |
| *Panulirus* | *argus* | 21.20 | -86.72 | sw | c | 35 | 1 | 11.4 | 0.325 | 1 | 38.7 | 0.55 | Rodriguez-Fuentes et al. 2017 |
| *Paramelita* | *nigroculus* | -33.97 | 18.42 | fw | c | 12 | NA | NA | NA | 1.4 | 35.3 | 0.17 | Buchanan et al. 1988 |
| *Penaeus* | *merguiensis* | NA | NA | sw | c | 21 | 0.02 | 5.3 | 0.42 | NA | NA | NA | Hoang et al. 2002 |
| *Penaeus* | *semisulcatus* | NA | NA | sw | c | 30 | NA | 6.4 | 0.27 | NA | NA | NA | Kir and Kumlu 2008 |
| *Portunus* | *pelagricus* | 22.12 | NA | sw | c | 21 | 0.2 | 11.6 | 0.37 | 0.2 | 42.3 | 0.22 | Qari 2014 |
| *Procambarus* | *clarkii* | 31.51 | -116.37 | fw | c | 15 | NA | NA | NA | 1 | 40.8 | 0.445 | Bückle Ramirez el al.1994 |
| *Saduria* | *entomon* | 60.37 | 22.05 | sw | c | 14 | NA | NA | NA | 0.2 | 27.5 | 0.11 | Lahdes et al. 1993 |
| *Acipenser* | *brevirostrum* | 32.89 | NA | fw | f | 7 | NA | NA | NA | 0.1 | 35.1 | 0.3 | Zieguewade et al. 2008 |
| *Amphyprion* | *ocellaris* | 17.45 | -99.48 | sw | f | 21 | 1 | 13.3 | 0.54 | 1 | 40.1 | 0.34 | Velazco et al. unpublished |
| *Anabas* | *testudineus* | 9.45 | NA | fw | f | 30 | 0.3 | 12.4 | 0.15 | 0.3 | 41.9 | 0.17 | Sarma et al. 2010 |
| *Apogon* | *novemfasciatus* | -5.46 | 123.79 | sw | f | 14 | 0.31 | 12.9 | 0.4 | 0.31 | 40.1 | 0.39 | Eme and Bennett 2009 |
| *Bathygobuis* | *fuscus* | -5.46 | 123.79 | sw | f | 14 | 0.31 | 9.2 | 0.29 | 0.31 | 42.4 | 0.14 | Eme and Bennett 2009 |
| *Bathygobuis* | *spp* | -5.46 | 123.79 | sw | f | 14 | 0.31 | 10.2 | 0.24 | 0.31 | 42.7 | 0.17 | Eme and Bennett 2009 |
| *Campostoma* | *anomalum* | 39.52 | -84.71 | fw | f | 14 | NA | NA | NA | 1 | 35.8 | 0.45 | Chagnon and Hlohowskyj 1989 |
| *Carassius* | *auratus* | 24.45 | NA | fw | f | 20 | 0.3 | 0.3 | 0.41 | 0.3 | 43.6 | 0.44 | Ford and Bettinger 2005 |
| *Catla* | *catla* | 20.44 | NA | fw | f | 30 | 0.3 | 13.9 | 0.18 | 0.3 | 42.7 | 0.24 | Das et al. 2004 |
| *Centropomus* | *undecimalis* | 21.18 | -90.03 | sw | f | 21 | 1 | 10.7 | 0.47 | 1 | 41.9 | 0.6 | Noyola et al. 2015 |
| *Cirrhinus* | *mrigala* | 20.63 | NA | fw | f | 30 | 0.3 | 12.1 | 0.19 | 0.3 | 43.1 | 0.08 | Das et al. 2004 |
| *Cottus* | *cognatus* | 41.88 | -87.63 | fw | f | 14 | NA | NA | NA | 0.5 | 29.4 | 0.43 | Otto and Rice 1977 |
| *Cyprinus* | *carpio* | 18.78 | -73.34 | fw | f | 30 | 0.3 | 8.4 | 0.18 | 0.3 | 42.9 | 0.32 | Chatterjee et al. 2004 |
| *Cyprinodon* | *macularius* | 32.06 | -111.67 | fw | f | 7 | NA | NA | NA | 0.5 | 43.2 | 0.3 | Lowe and Heath 1969 |
| *Cyprinodon* | *nevadensis* | 36.24 | -116.86 | fw | f | 7 | 0.03 | 0.8 | 0.17 | 0.03 | 42.7 | 0.14 | Feldmeth et al. 1974 |
| *Cyprinodon* | *spp* | 36.51 | -116.98 | fw | f | 7 | NA | NA | NA | 0.3 | 43.6 | 0.23 | Otto and Gerking 1973 |
| *Cyprinodon* | *variegatus* | 26.07 | -97.16 | fw | f | 30 | 0.1 | 0.6 | 0.32 | 0.1 | 44.2 | 0.29 | Bennett and Beitinger 1997 |
| *Danio* | *rerio* | 21.37 | NA | fw | f | 11 | 0.3 | 6.2 | 0.44 | 0.3 | 41.7 | 0.25 | Cortemeglia and Beitinger 2011 |
| *Dascyllus* | *aruanus* | -5.46 | 123.79 | sw | f | 14 | 0.31 | 12 | 0.33 | 0.31 | 40.5 | 0.39 | Eme and Bennett 2009 |
| *Dasyatis* | *sabina* | 29.81 | -85.35 | sw | f | 20 | 0.3 | 0.8 | 0.41 | 0.3 | 43.2 | 0.31 | Eme and Bennett 2009 |
| *Dicentrarchus* | *labrax* | 36.76 | NA | sw | f | 35 | 0.3 | 4.1 | 0.27 | 0.3 | 36 | 0.28 | Dülger et al. 2012 |
| *Elacatinus* | *oceanops* | 16.76 | -88.14 | sw | f | 365 | 0.5 | 14.7 | 0.29 | 0.5 | 35.5 | 0.46 | DiSanto and Lobel 2017 |
| *Elacatinus* | *lobeli* | 16.76 | -88.14 | sw | f | 365 | 0.5 | 13.8 | 0.4 | 0.5 | 39.1 | 0.52 | DiSanto and Lobel 2017 |
| *Fundulus* | *heteroclitus* | 42.92 | -70.80 | sw | f | 21 | 0.3 | -1.1 | 0.29 | 0.3 | 41.8 | 0.41 | Fange et al. 2006 |
| *Fundulus* | *heteroclitus* | 31.15 | -81.30 | sw | f | 21 | 0.3 | -1.1 | 0.35 | 0.3 | 42.5 | 0.36 | Fange et al. 2006 |
| *Gambusia* | *affinis* | 40.81 | -111.95 | fw | f | 30 | NA | NA | NA | 0.3 | 42.1 | 0.36 | Otto 1973 |
| *Gambusia* | *affinis* | 33.00 | -109.90 | fw | f | 30 | NA | NA | NA | 0.3 | 43.2 | 0.34 | Otto 1973 |
| *Gasterosteus* | *aculeatus* | 38.57 | NA | sw | f | NA | NA | NA | NA | 0.25 | 34.6 | 0.27 | Helmet and Baski 1976 |
| *Gibbonsia* | *elegans* | 34.45 | -120.47 | sw | f | 7 | 0.05 | 3 | 0.24 | 0.07 | 31.6 | 0.14 | Davis 1977 |
| *Gibbonsia* | *montereyensis* | 35.64 | -121.19 | sw | f | 7 | 0.05 | 2.1 | 0.17 | 0.07 | 29.3 | 0.22 | Davis 1977 |
| *Gila* | *bicolor* | 35.65 | -117.66 | fw | f | 7 | 0.14 | 2.8 | 0.37 | 0.14 | 36.2 | 0.23 | McClanahan et al. 1986 |
| *Gobionotothen* | *gibberifrons* | -77.50 | 165.00 | sw | f | 7 | NA | NA | NA | 0.3 | 17.9 | 0.31 | Bilyk and Devries 2011 |
| *Horabagrus* | *brachysoma* | 21.85 | NA | fw | f | 30 | 0.3 | 13.2 | 0.22 | 0.3 | 42.8 | 0.37 | Dalvi et al. 2009 |
| *Ictalurus* | *punctatus* | 36.73 | NA | fw | f | 20 | 0.3 | 2.7 | 0.71 | 0.3 | 40.3 | 0.39 | Currie et al. 1998 |
| *Ictalurus* | *punctatus* | 32.14 | -115.28 | fw | f | 30 | NA | NA | NA | 1 | 42.5 | 0.62 | Diaz et al. 1999 |
| *Labeo* | *rohita* | 18.90 | NA | fw | f | 30 | 0.3 | 13.7 | 0.19 | 0.3 | 42.9 | 0.24 | Das et al. 2004 |
| *Lepomis* | *gibbosus* | 46.29 | -119.28 | fw | f | 14 | NA | NA | NA | 0.3 | 35.1 | 0.5 | Becker and Genoway 1979 |
| *Lepomis* | *macrochirus* | 33.29 | -81.73 | fw | f | 14 | NA | NA | NA | 1 | 40.9 | 0.46 | Holland et al. 1974 |
| *Limia* | *melanonotata* | 18.22 | -71.10 | fw | f | 10.5 | NA | NA | NA | 0.3 | 43 | 0.31 | Haney and Walsh 2003 |
| *Liza* | *viagiensis* | -5.46 | 123.79 | sw | f | 14 | 0.31 | 9.9 | 0.42 | 0.31 | 44.5 | 0.3 | Eme and Bennett 2009 |
| *Lutjanus* | *gutattus* | 17.45 | -99.48 | sw | f | 21 | 1 | 10.8 | 0.7 | 1 | 40.9 | 0.37 | Larios-Soriano 2014 |
| *Lycodichthys* | *dearbornii* | -77.50 | 165.00 | sw | f | 21 | NA | NA | NA | 0.3 | 15.4 | 0.34 | Bilyk and Devries 2011 |
| *Micropterus* | *salmoides* | 34.70 | NA | fw | f | 20 | 0.3 | 3.2 | 0.75 | 0.3 | 38.5 | 0.31 | Currie et al. 1998 |
| *Notolabrus* | *celidotus* | -43.60 | 172.84 | sw | f | 28 | 0.03 | 3.5 | 0.47 | 0.03 | 32.4 | 0.38 | Hooper 2009 |
| *Notothenia* | *coriiceps* | -77.50 | 165.00 | sw | f | 14 | NA | NA | NA | 0.3 | 17.4 | 0.2 | Bilyk and Devries 2011 |
| *Ocyurus* | *crysurus* | 21.18 | -90.03 | sw | f | 21 | 1 | 11.8 | 0.31 | 1 | 38.7 | 0.35 | Noyola et al. 2015 |
| *Oncorhynchus* | *apache* | 33.69 | -109.79 | fw | f | 14 | NA | NA | NA | 0.02 | 29.4 | 0.09 | Lee and Rinne 2011 |
| *Oncorhynchus* | *clarkii* | 48.23 | NA | sw | f | NA | NA | NA | NA | 0.4 | 29.9 | 0.23 | Heath 1963 |
| *Oncorhynchus* | *gilae* | 33.30 | -107.96 | fw | f | 14 | NA | NA | NA | 0.02 | 29.6 | 0.13 | Lee and Rinne 2011 |
| *Oncorhynchus* | *kisutch* | 46.29 | -119.28 | fw | f | 14 | NA | NA | NA | 0.3 | 28.7 | 0.34 | Becker and Genoway 1979 |
| *Oncorhynchus* | *mykiss* | 38.30 | NA | fw | f | 20 | 0.3 | 0 | 0.2 | 0.3 | 29.8 | 0.18 | Currie et al. 1998 |
| *Pachycara* | *branchycephalum* | -77.50 | 165.00 | sw | f | 21 | NA | NA | NA | 0.3 | 17.2 | 0.46 | Bilyk and Devries 2011 |
| *Pagothenia* | *borchgrevinki* | -77.50 | 165.00 | sw | f | 21 | NA | NA | NA | 0.3 | 15.2 | 0.54 | Bilyk and Devries 2011 |
| *Pimephales* | *promelas* | 33.21 | -97.13 | fw | f | 7 | NA | NA | NA | 0.3 | 40.4 | 0.45 | Richard and Beitinger 1995 |
| *Pangasius* | *pangasius* | 22.57 | NA | fw | f | 30 | 0.3 | 12.4 | 0.6 | 0.3 | 44.1 | 0.17 | Debnath et al. 2006 |
| *Poecilia* | *sphenops* | 17.03 | -96.50 | fw | f | 30 | 1 | 7.5 | 0.3 | 1 | 43 | 0.3 | Hernandez and Bückle 2002 |
| *Prochilodus* | *scrofa* | -22.00 | -22.00 | fw | f | 30 | 0.1 | 6.5 | 0.41 | 0.1 | 42.6 | 0.47 | Barrionuevo and Fernandes 1995 |
| *Pterophyllum* | *scalare* | -2.17 | NA | fw | f | 30 | NA | NA | NA | 1 | 41.2 | 0.41 | Perez et al. 2003 |
| *Rhinichthys* | *osculus* | 43.28 | -110.02 | fw | f | 20 | NA | NA | NA | 0.45 | 34.6 | 0.03 | Kaya et al. 1992 |
| *Rhinichthys* | *osculus* | 42.87 | -109.86 | fw | f | 20 | NA | NA | NA | 0.45 | 34.9 | 0.33 | Kaya et al. 1992 |
| *Salvelinus* | *fontinalis* | 33.93 | -109.59 | fw | f | 14 | NA | NA | NA | 0.02 | 29.8 | 0.11 | Lee and Rinne 2011 |
| *Salmo* | *salar* | 54.24 | -3.00 | fw | f | 14 | NA | NA | NA | 0.033 | 32.7 | 0.02 | Elliott and Elliot 1995 |
| *Salmo* | *trutta* | 33.93 | -109.59 | fw | f | 14 | NA | NA | NA | 0.02 | 29.9 | 0.09 | Lee and Rinne 2011 |
| *Sebastiscus* | *marmoratus* | 23.57 | NA | sw | f | 14 | 0.08 | 4.9 | 0.31 | 0.08 | 32.8 | 0.31 | Kita et al. 1996 |
| *Thymallus* | *arcticus* | 45.66 | -112.90 | fw | f | 14 | NA | NA | NA | 0.4 | 29.3 | 0.25 | Lohr et al. 2011 |
| *Tilapia* | *UNAM* | 20.07 | -97.05 | fw | f | 21 | 1 | 8.22 | 0.67 | 1 | 43.4 | 0.36 | Medina Romo 2018 |
| *Tor* | *putitora* | 21.95 | NA | fw | f | 30 | NA | NA | NA | 0.3 | 41.8 | 0.47 | Aktar et al. 2013 |
| *Trematomus* | *bernacchii* | -77.50 | 165.00 | sw | f | 21 | NA | NA | NA | 0.3 | 15 | 0.24 | Bilyk and Devries 2011 |
| *Trematomus* | *hansoni* | -77.50 | 165.00 | sw | f | 21 | NA | NA | NA | 0.3 | 15.4 | 0.39 | Bilyk and Devries 2011 |
| *Trematomus* | *pennelii* | -77.50 | 165.00 | sw | f | 21 | NA | NA | NA | 0.3 | 15.4 | 0.53 | Bilyk and Devries 2011 |
| *Xiphophorus* | *maculatus* | 21.90 | NA | fw | f | 7 | 0.13 | 9.6 | 0.39 | 0.13 | 41.5 | 0.11 | Prodocimo and Freire 2001 |

**References:**

Akhtar, M.S., Pal, A.K., Sahu, N.P., Ciji, A., Mahanta, P.C. (2013) Thermal tolerance, oxygen consumption and haemato-biochemical variables of *Tor putitora* juveniles acclimated to five temperatures, *Fish Physiology and Biochemistry*, *39*, 1387–1398, doi: 10.1007/s10695-013-9793-7

Barrionuevo, W.R., Femandes, M.N. (1995) Critical thermal maxima and minima for curimbatá, *Prochilodus scrofa* Steindachner, of two different sizes, *Aquaculture Research*, 26, 447–450, doi: 10.1111/j.1365-2109.1995.tb00934.x

Becker, C.D., Genoway, R.G. (1979) Evaluation of the critical thermal maximum for determining thermal tolerance of freshwater fish, *Environmental Biology of Fishes*, 4, 245–256, doi: 10.1007/BF00005481

Bennett, W.A., Beitinger, T.L. (1997) Temperature Tolerance of the Sheepshead Minnow, *Cyprinodon variegatus*, *Copeia*, 1, 77-87

Bilyk, K.T., DeVries, A.L. (2011) Heat tolerance and its plasticity in Antarctic fishes, *Comparative Biochemistry and Physiology, Part A*, 158, 382–390, doi: 10.1016/j.cbpa.2010.12.010

Buchanan, J.A., Stewart, B.A., Davies, B.R. (1988) Thermal acclimation and tolerance to lethal high temperature in the mountain stream amphipod *Paramelita nigroculus* (Barnard), *Comparative Biochemistry and Physiology Part a: Physiology*, *89*(3), 425–431, doi: 10.1016/0300-9629(88)91051-1

Bückle Ramírez, L.F., Diaz Herrera, F., Correa Sandoval, F., Barón Sevilla, B., Hernández Rodriguez, M. (1994) Diel thermoregulation of the crawfish *Procambarus Clarkii* (crustacea, cambaridae), *Journal of Thermal Biology*, *19*(6), 419–422, doi: 10.1016/0306-4565(94)90041-8

Camacho, J., Qadri, S.A., Wang, H., Worden, M.K. (2006) Temperature acclimation alters cardiac performance in the lobster *Homarus americanus*, *Journal of Comparative Physiology A*, 192,1327-1334, doi: 10.1007/s00359-006-0162-1

Chagnon, N., Hlohowskyj, I. (1989) Effects of phenol exposure on the thermal tolerance ability of the central Stoneroller Minnow. *Bulletin of Environmental Contamination and Toxicology*, 42, 614–619, doi: 10.1007/BF01700246

Chatterjee, N.Pal, A.K., Manush, S.M., Das, T., Mukherjee, S.C. (2004) Thermal tolerance and oxygen consumption of *Labeo rohita* and *Cyprinus carpio* early fingerlings acclimated to three different temperatures, *J.Thermal Biol*., 29, 265-270, doi: 10.1016/j.jtherbio.2004.05.001

Cheng, S.-Y., Chen, C.-S., Chen, J.-C. (2012) Salinity and temperature tolerance of brown-marbled grouper *Epinephelus fuscoguttatus*. *Fish Physiology and Biochemistry*, 39, 277–286, doi: 10.1007/s10695-012-9698-x

Claussen, D.L. (1980) Thermal acclimation in the crayfish, *Orconectes rusticus* and *O.virilis*. *Comparative Biochemistry and Physiology Part A*, 66, 377–384, doi: 10.1016/0300-9629(80)90183-8

Cortemeglia, C., Beitinger, T.L. (2011) Temperature tolerances of wild-type and red transgenic zebra danios, *Trans.Amer.Fish.Soc*.,  6, 1431-1437, doi: 10.1577/T04-197.1

Cuculescu, M., Hyde, D., Bowler, K. (1998) Thermal tolerance of two species of marine crab, *Cancer pagurus* and *Carcinus maenas*. *Journal of Thermal Biology*, 23, 107–110, doi: 10.1016/S0306-4565(98)00008-4

Cumillaf, J.P., Blanc, J., Paschke, K., Gebauer, P., Diaz, F., Re, D., et al. (2016) Thermal biology of the sub-polar-temperate estuarine crab *Hemigrapsus crenulatus* (Crustacea: Decapoda: Varunidae), *Biology Open*, 5, 220–228, doi: 10.1242/bio.013516

Currie, R.J., Bennett, W.A., Beitinger, T.L. (1998) Critical thermal minima and maxima of three freshwater game-fish species acclimated to constant temperatures, *Environmental Biology of Fishes*, 51, 187–200, doi: 10.1023/A:1007447417546

Dalvi, R.S., Pal, A.K., Tiwari, L.R., Das, T., Baruah, K. (2009) Thermal tolerance and oxygen consumption rates of the catfish *Horabagrus brachysoma* (Günther) acclimated to different temperatures. *Aquaculture*, 295, 116-119, doi: 10.1016/j.aquaculture.2009.06.034

Das T., Pal, A.K., Chakraborty, S.K., Manush, S.M., Chatterjee, N., Mukherjee, S.C. (2004) Thermal tolerance and oxygen consumption of Indian Major Carps acclimated to four temperatures, *J.Thermal Biol*., 29, 157-163, doi: 10.1016/j.jtherbio.2004.02.001

Davis, B.J. (1977) Distribution and temperature adaptation in the teleost fish genus *Gibbonsia*. *Marine Biology*, 42, 315-320, doi: 10.1007/BF00402193

Debnath, D., Pal, A.K., Sahu, N.P., Baruah, K., Yengkokpam, S., Das, T., Manush, S.M. (2006) Thermal tolerance and metabolic activity of yellowtail catfish *Pangasius pangasius* (Hamilton) advanced fingerlings with emphasis on their culture potential, *Aquaculture*, 258, 606–610, doi: 10.1016/j.aquaculture.2006.04.037

Di Santo, V., Lobel, P.S. (2017) Body size and thermal tolerance in tropical gobies. *Journal of Experimental Marine Biology and Ecology*, 487, 11–17, doi: 10.1016/j.jembe.2016.11.007

Díaz, F., Re, A.D., Galindo-Sánchez, C.E., Carpizo-Ituarte, E., Perez-Carrasco, L., González, M., et al. (2017) Preferred temperature, critical thermal maximum, and metabolic response of the black sea urchin *Arbacia stellate* (Blainville, 1825; Gamelin, 1791), *Journal of Shellfish Research*, 36, 219–225, doi: 10.2983/035.036.0124

Díaz, F., Re, A.D., Salas, A., Galindo-Sanchez, C.E., Gonzalez, M.A., Sanchez, A., Rosas, C. (2015) Behavioral thermoregulation and critical thermal limits of giant keyhole limpet *Megathura crenulata* (Sowerby 1825) (Mollusca; Vetigastropoda), *J.Thermal Biol*., 54, 133-138, doi: 10.1016/j.jtherbio.2013.05.007

Díaz, F., Salas, A., Re, A.D., González, M., Reyes, I. (2011) Thermal preference and tolerance of *Megastrea (Lithopoma) undosa*(Wood 1828) (Gastropoda: Turbinidae) *J.Thermal Biol*., 36, 34–37.doi: 10.1016/j.jtherbio.2010.10.004

Díaz, F., Re, A.D., Medina, Z., Re, G., Valdez, G., Valenzuela, F. (2006) Thermal preference and tolerance of green abalone *Haliotis fulgens*(Philippi, 1845) and pink abalone *Haliotis corrugata*(Gray, 1828) *Aquacultur.Res*., 37, 877-884, doi: 10.1111/j.1365-2109.2006.01506.x

Diaz, F., Re, A.D., Sierra, E., Amador, G. (2004) Behavioral thermoregulation and critical limits applied to culture of red claw crayfish *Cherax quadricarinatus* (Von Martens), *Freshwater Crayfish*, 14, 90-98

Diaz, F., Sierra, E., Re, A.D., Rodriguez, L. (2002) Behavioural thermoregulation and critical thermal limits of *Macrobrachium acanthurus* (Wiegman), *Journal of Thermal Biology*, 27, 423-428, doi: 10.1016/S0306-4565(02)00011-6

Dı́az, F., del Rı́o-Portı́lla, M.A., Sierra, E., Aguilar, M., Re-Araujo, A.D. (2000) Preferred temperature and critical thermal maxima of red abalone *Haliotis rufescens*, *Journal of Thermal Biology*, 25, 257-261, doi: 10.1016/S0306-4565(99)00032-7

Dı́az, F., Bückle, L.F. (1999) Effect of the critical thermal maximum on the preferred temperatures of *Ictalurus punctatus* exposed to constant and fluctuating temperatures, *Journal of Thermal Biology*, 24, 155-160, doi: 10.1016/S0306-4565(99)00005-4

Díaz, F., Uribe, E.S., Ramirez, L., Mora, A.G. (1998) Critical thermal maxima and minima of *Macrobrachium rosenbergii* (Decapoda : Palaemonidae), *Journal of Thermal Biology*, 23, 381-385, doi: 10.1016/S0306-4565(98)00029-1

Dülger, N., Kumlu, M., Türkmen, S., Ölçülü, A., Eroldoğan, O.T., Yılmaz, H.A., Öçal, N. (2012) Thermal tolerance of European Sea Bass (*Dicentrarchus labrax*) juveniles acclimated to three temperature levels, *J.Thermal Biol*., 37, 79-82, doi: 10.1016/j.jtherbio.2011.11.003.

Elliott, J.M., Elliott, J.A. (1995) The effect of the rate of temperature increase on the critical thermal maximum for Parr of Atlantic Salmon and Brown Trout. *Journal of Fish Biology*, 47, 917–919, doi: 10.1111/j.1095-8649.1995.tb06014.x

Eme, J., Bennett, W.A. (2009) Acute temperature quotient responses of fishes reflect their divergent thermal habitats in the Banda Sea, Sulawesi, Indonesia.Aus.J.Zool., 57, 357-362.doi: 10.1071/ZO09081

Fangue, N.A. (2006) Intraspecific variation in thermal tolerance and heat shock protein gene expression in common killifish, *Fundulus heteroclitus*. *The Journal of Experimental Biology*, 209, 2859–2872, doi: 10.1242/jeb.02260

Ford, T., Beitinger, T.L. (2005) Temperature tolerance in the goldfish, *Carassius auratus*. *Journal of Thermal Biology*, *30*, 147–152.

Haney, D.C., Walsh, S.J. (2003) Influence of salinity and temperature on the physiology of *Limia melanonotata* (Cyprinodontiforme: Poeciliidae): a search for abiotic factors limiting insular distribution in Hispaniola, *Caribbean Journal of Science*, 39, 327-337

Heath, W.G. (1963) Thermoperiodism in Sea-run Cutthroat Trout (*Salmo clarki clarki*), *Science*.25, 486-4888.

Hernandez, R.M., Bückle, R. (2002) Temperature tolerance polygon of *Poecilia sphenops* Valenciennes (Pisces : Poeciliidae), *Journal of Thermal Biology*, 27, 1-5, doi: 10.1016/S0306-4565(01)00008-0

Hoang, T., Lee, S.Y., Keenan, C.P., Marsden, G.E. (2002) Effect of temperature on spawning of *Penaeus merguiensis*, *Journal of Thermal Biology*, 27, 433-437, doi: 10.1016/S0306-4565(02)00013-X

Holland, W.E., Smith, M.E., Gibbons, J.W., Brown, D.H. (1974) Thermal tolerances of fish from a reservoir receiving heated effluent from a nuclear reactor, *Physiol.Zool*., 47, 110-118

Hooper, J.K. (2009) The effect of temperature change on the New Zealand marine fish, *Notolabrus celidotus*, master thesis

Kaya, C., Brussard, P.F., Cameron, D.G., Vyse, E.R. (1992) Biochemical genetics and thermal tolerances of Kendall Warm Springs dace (*Rhinichthys osculus thermalis*) and Green River Speckled dace (*R.o.yarrowi*), *Copeia*, 2, 528-535

Kita, J., Tsuchida, S., Setoguma, T. (1996) Temperature preference and tolerance, and oxygen consumption of the marbled rockfish, *Sebastiscus marmoratus*. *Marine Biology*, 125, 467-471, doi: 10.1007/BF00353259

Kιr, M., Kumlu, M. (2008) Effect of temperature and salinity on low thermal tolerance of *Penaeus semisulcatus* (Decapoda: Penaeidae) *Aquacultur.Res*., 39, 1101-1106, doi: 10.1111/j.1365-2109.2008.01973.x

Kumlu, M., Türkmen, S., Kumlu, M. (2010) Thermal tolerance of *Litopenaeus vannamei* (Crustacea: Penaeidae) acclimated to four temperatures, *J.Thermal Biol*., 35, 305-308, doi : 10.1016/j.jtherbio.2010.06.009.

Lagerspetz, K.Y.H., Bowler, K. (1993) Variation in heat tolerance in individual *Asellus aquaticus* during thermal acclimation. *Journal of Thermal Biology*, 18, 137–143, doi: 10.1016/0306-4565(93)90027-Q

Lahdes, E.O., Kivivuori, L.A., Lehti-Koivunen, S.M. (1993) Thermal tolerance and fluidity of neuronal and branchial membranes of an antarctic amphipod (*Orchomene plebs*); a comparison with a baltic isopod (*Saduria entomon*), *Comparative Biochemistry and Physiology Part A*, 105, 463–470, doi: 10.1016/0300-9629(93)90420-9

Lee, R.M., Rinne, J.N. (2011) Critical thermal maxima of five trout species in the southwestern United States, *Trans.Amer.Fish.Soc*.,  109, 632-635, doi: 10.1577/1548-8659(1980)109<632:CTMOFT>2.0.CO;2

Lohr, S.C., Byorth, P.A., Kaya, C.M., Dwyer, W.P. (2011) High-temperature tolerances of fluvial arctic grayling and comparisons with summer river temperatures of the Big Hole River, Montana, *Trans.Amer.Fish.Soc*., 125, 933-939, doi: 10.1577/1548-8659(1996)125<0933:HTTOFA>2.3.CO;2

Lowe, C., Heath, W.G. (1969) Behavioral and physiological responses to temperature in the desert pupfish *Cyprinodon macularius, Physiol.Zool*., 42, 53-59

Lugo, P., Díaz, F., Re, A.D., Olivares, F., González, R., Dueñas, S., Licea, A. (2016) Thermoregulatory behaviour and thermal tolerance of three species of Conidae in the Eastern Pacific and Gulf of California coasts of Baja California, Mexico, *Mollusc.Res*., 36, 247-254, doi: 10.1080/13235818.2016.1172545

Magozzi, S., Calosi, P. (2014) Integrating metabolic performance, thermal tolerance, and plasticity enables for more accurate predictions on species vulnerability to acute and chronic effects of global warming, *Global Change Biology*, 21, 181-194, doi: 10.1111/gcb.12695

McClanahan, L.L., Feldmeth, C.R., Jones, J., Soltz, D.L. (1986) Energetics, salinity and temperature tolerance in the mohave tui chub, *Gila bicolor mohavensis*, *Copeia*, 1, 45-52

Mcgaw I.J. (2003) Behavioral thermoregulation in *Hemigrapsus nudus*, the amphibious purple shore crab, *Biol.Bull*., 204, 38-49

Medina Romo, E.Z. (2018) Caracterización fisiológica y metabólica de la tilapia tetra híbrida Pargo UNAM a diferentes temperaturas y salinidades de aclimatación, doctoral thesis.

Morritt, D., Ingólfsson, A. (2000) Upper thermal tolerances of the beachflea *Orchestia gammarellus* (Pallas) (Crustacea: Amphipoda: Talitridae) associated with hot springs in Iceland, *Journal of Experimental Marine Biology and Ecology*, 255, 215–227, doi: 10.1016/S0022-0981(00)00299-9

Noyola Regil, J., Mascaro, M., Díaz, F., Re, A.D., Sánchez-Zamora, A., Caamal-Monsreal, C., Rosas, C. (2015) Thermal biology of prey (*Melongena corona bispinosa*, *Strombus pugilis*, *Callinectes similis*, *Libinia dubia*) and predators (*Ocyurus chrysurus*, *Centropomus undecimalis*) of *Octopus maya* from the Yucatan Peninsula, *Journal of Thermal Biology*, 53, 151–161. doi: 10.1016/j.jtherbio.2015.11.001

Noyola, J., Caamal-Monsreal, C., Díaz, F., Re, D., Sánchez, A., Rosas, C. (2013). Thermopreference, tolerance and metabolic rate of early stages juvenile *Octopus maya* acclimated to different temperatures, *Journal of Thermal Biology*, 38, 14–19. doi: 10.1016/j.jtherbio.2012.09.001

Otto, R.G., Rice, J.O. (1977) Responses of a freshwater sculpin (*Cottus cognatus gracilis*) to temperature.*Trans.Amer.Fish.Soc*., 106: 89-94.doi:b10.1577/1548-8659(1977)106<89:ROAFSC>2.0.CO;2

Otto, R.G., Gerking, S.D. (1973) Heat tolerance of a death valley pupfish (Genus *Cyprinodon*), *Physiol.Zool*., 46, 43-49

Otto, R.G. (1973) Temperature tolerance of the mosquitofish, *Gambmia affinis* (Baird and Girard), *Journal of Fish Biology*, 5, 575–585, doi: 10.1111/j.1095-8649.1973.tb04490.x

Padilla-Ramírez, S., Díaz, F., Re, A.D., Galindo-Sanchez, C.E., Sanchez-Lizarraga, A.L., Nuñez-Moreno, L.A., et al. (2015) The effects of thermal acclimation on the behavior, thermal tolerance, and respiratory metabolism in a crab inhabiting a wide range of thermal habitats (*Cancer antennarius* Stimpson, 1856, the red shore crab), *Marine and Freshwater Behaviour and Physiology*, 48, 89–101, doi: 10.1080/10236244.2015.1019212

Pérez, E., Dı́az, F., Espina, S. (2003) Thermoregulatory behavior and critical thermal limits of the angelfish *Pterophyllum scalare* (Lichtenstein) (Pisces: Cichlidae), *Journal of Thermal Biology*, 28, 531–537, doi: 10.1016/S0306-4565(03)00055-X

Prodocimo, V., Freire, C.A. (2001) Critical thermal maxima and minima of the platyfish *Xiphophorus maculatus* Günther (Poecillidae, Cyprinodontiformes): a tropical species of ornamental freshwater fish, *Revista Brasileira De Zoologia*, 18, 97-106, doi: 10.1590/S0101-81752001000500007

Qari, S. (2014) Heat shock response of the blue crab *Portunus pelagicus*: thermal stress and acclimation, *Journal of coastal life medicine*, 2, 609-613, doi: 10.12980/JCLM.2.2014JCLM-2014-0057

Ravaux, J., Léger, N., Rabet, N., Morini, M., Zbinden, M., Thatje, S., Shillito, B. (2012) Adaptation to thermally variable environments: capacity for acclimation of thermal limit and heat shock response in the shrimp *Palaemonetes varians*, *Journal of Comparative Physiology B*, 182, 899-907, doi: 10.1007/s00360-012-0666-7

Re, A.D., Díaz, F., Salas-Garza, A., Gonzalez, M., Cordero, V., Galindo-Sánchez, C.E., et al. (2013) Thermal preference, tolerance and temperature-dependent respiration in the California sea hare *Aplysia californica*, *Agricultural Sciences*, 4, 46–52, doi: 10.4236/as.2013.46A007

Re, A.D., Diaz, F., Sierra, E., Rodríguez, J., Perez, E. (2005) Effect of salinity and temperature on thermal tolerance of brown shrimp *Farfantepenaeus aztecus* (Ives) (Crustacea, Penaeidae), *J.Thermal Biol*, 30, 618-622, doi: 10.1016/j.jtherbio.2005.09.004

Reiser, S., Herrmann, J.-P., Neudecker, T., Temming, A. (2013) Lower thermal capacity limits of the common brown shrimp (*Crangon crangon*, L.) *Marine Biology*, 161, 447–458, doi: 10.1007/s00227-013-2350-1

Richards, V.L., Beitinger, T.L. (1995) Reciprocal influences of temperature and copper on survival of fathead minnows, *Pimephales* *promelas*, *Bulletin of Environmental Contamination and Toxicology*, 55, 230–236, doi: 10.1007/BF00203014

Feldmeth, R.C., Stone, E.A., Brown, J.H. (1974) An increased scope for thermal tolerance upon acclimating pupfish (*Cyprinodon*) to cycling temperatures, *Journal of Comparative Physiology*, 89, 39–44, doi: 10.1007/BF00696161

Rodríguez-Fuentes, G., Murúa-Castillo, M., Díaz, F., Rosas, C., Caamal-Monsreal, C., Sánchez, A., et al. (2017). Ecophysiological biomarkers defining the thermal biology of the Caribbean lobster *Panulirus argus*, *Ecological Indicators*, 78, 192–204. doi: 10.1016/j.ecolind.2017.03.011

Rodriguez, M.H., Ramirez, L. (1997) Thermal preference area for *Macrobrachium tenellum* in the context of global climatic change, *Journal of Thermal Biology*, 22, 309–313, doi: 10.1016/S0306-4565(97)00027-2

Salas, A., Díaz, F., Re, A.D., Galindo-Sánchez, C.E., Sanchez-Castrejon, E., González, M., et al. (2014) Preferred temperature, thermal tolerance, and metabolic response of *Tegula regina* (Stearns, 1892), *Journal of Shellfish Research*, 33, 239–246, doi: 10.2983/035.033.0123

Sarma, K., Pal, A.K., Ayyappan, S., Das, T., Manush, S.M., Debnath, D., Baruah, K. (2008) Acclimation of *Anabas testudineus* (Bloch) to three test temperatures influences thermal tolerance and oxygen consumption, *Fish Physiology and Biochemistry*, 36, 85-90, doi: 10.1007/s10695-008-9293-3

Selvakumar, S., Geraldine, P. (2005) Heat shock protein induction in the freshwater prawn *Macrobrachium malcolmsonii*: Acclimation-influenced variations in the induction temperatures for Hsp70, *Comparative Biochemistry and Physiology-Part A*, 140, 209–215, doi: 10.1016/j.cbpb.2005.01.008

Ziegeweid, J.R., Jennings, C.A., Peterson, D.L., Black, M.C. (2008) Effects of salinity, temperature, and weight on the survival of young‐of‐year shortnose sturgeon.*Trans.Amer.Fish.Soc*., 137: 1490-1499, doi: 10.1577/T07-046.1

Zúñiga, O., Olivares, A., Rojo, M., Chimal, M.E., Díaz, F., Uriarte, I., Rosas, C. (2013) Thermoregulatory behavior and oxygen consumption of *Octopus mimus* paralarvae: The effect of age, *Journal of Thermal Biology*, 38, 86–91, doi: 10.1016/j.jtherbio.2012.11.003
