# Supplementary material for: Comparison of Aerobic Scope for Metabolic Activity in Aquatic Ectotherms With Temperature Related Metabolic Stimulation: A Novel Approach for Aerobic Power Budget
Source: Front Physiol. 2018 Oct 22;9:1438. doi: 10.3389/fphys.2018.01438 (PMC6204536; doi:10.3389/fphys.2018.01438)
Supplement: Table S2 — Values obtained from LMR and HMR measured at TIMR min and TIMR max conditions for 5 min for Centropomus undecimalis (Snook), Ocyurus chrysurus (Yellow tail) and Amphyprion ocellaris (clownfish). [file Table_2.docx]

| **sT2.** Q_10_ values obtained from LMR and HMR measured at TIMR min and TIMR max conditions for 5 min for *Centropomus undecimalis* (snook), *Ocyurus chrysurus* (yellow tail) and *Amphyprion ocellaris* (clownfish). | | | |
| --- | --- | --- | --- |
| **°C** | **°C** | **°C** | **Q10** |
| *Centropomus undecimalis* | |  |  |
| 18 | 12.1 | 33.3 | 1.51 |
| 22 | 12.1 | 34.1 | 1.83 |
| 26 | 13.0 | 34.5 | 1.87 |
| 28 | 14.3 | 36.2 | 1.91 |
| 30 | 14.4 | 36.9 | 1.87 |
| 32 | 16.5 | 37.0 | 2.01 |
| 35 | 18.7 | 38.7 | 1.70 |
| *Ocyurus chrysurus* | |  |  |
| 20 | 12.6 | 33.0 | 1.32 |
| 22 | 13.0 | 33.9 | 1.41 |
| 26 | 16.3 | 34.0 | 1.97 |
| 30 | 17.0 | 35.0 | 1.79 |
| 32 | 18.0 | 36.0 | 1.90 |
| *Amphiprion ocellaris* | |  |  |
| 20 | 14.8 | 31.4 | 1.60 |
| 22 | 16.5 | 32.5 | 2.76 |
| 26 | 18.0 | 33.6 | 2.91 |
| 30 | 20.2 | 35.7 | 2.96 |
| 32 | 22.0 | 35.8 | 3.10 |
| 35 | 23.7 | 36.0 | 1.51 |
