# Supplementary figures and images for: Comparison of Aerobic Scope for Metabolic Activity in Aquatic Ectotherms With Temperature Related Metabolic Stimulation: A Novel Approach for Aerobic Power Budget
Source: Front Physiol. 2018 Oct 22;9:1438. doi: 10.3389/fphys.2018.01438 (PMC6204536; doi:10.3389/fphys.2018.01438)

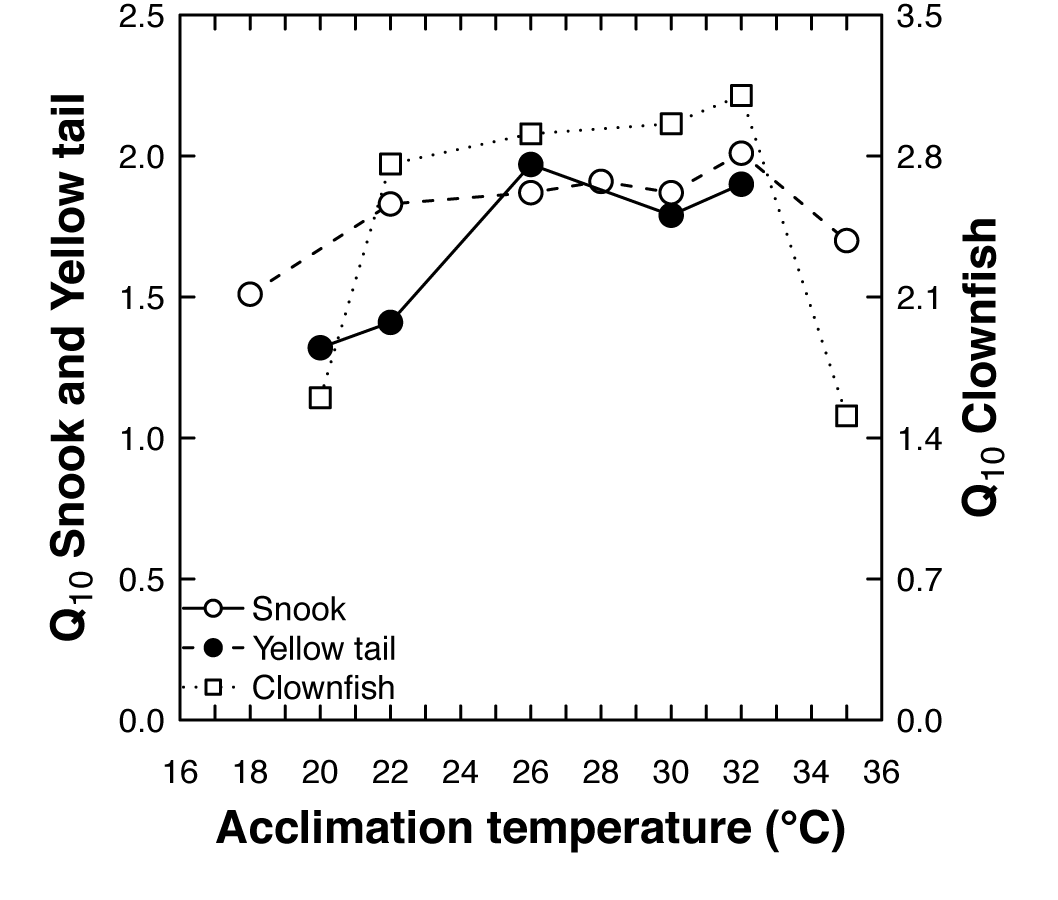

Supplement: Figure S1 — Effect of acclimation temperature in Q10 values obtained using data of oxygen consumption (Low and high metabolic rates, i.e., LMR and HMR) of the three fish species exposed at the temperatures used to induce metabolic rates (TIMR min and max, respectively) at each acclimation temperature. Note that data of clownfish where placed in the secondary axis to improve its visualization. [file Image_1.TIF]
